# Catalysis in Silver Nanocube Formation: The Role of Iron Ions in Non-Polar Solvents

**DOI:** 10.1021/acsnanoscienceau.5c00103

**Published:** 2025-10-09

**Authors:** Maximilian Joschko, Moritz Schattmann, Deniz Grollmusz, Tobias Reich, Christina Graf

**Affiliations:** † Hochschule Darmstadt - University of Applied Sciences, Fachbereich Chemie- und Biotechnologie, Stephanstr. 7, D-64295 Darmstadt, Germany; ‡ EUt+ Institute of Nanomaterials & Nanotechnologies EUTINN, European University of Technology, European Union, https://www.univ-tech.eu/eutinn; § Department of Chemistry − Nuclear Chemistry, Johannes Gutenberg-Universität Mainz, Fritz-Strassmann-Weg 2, D-55128 Mainz, Germany

**Keywords:** silver, nanoparticles, nanocubes, catalysis, metal−organic synthesis, hot-injection synthesis

## Abstract

Plasmonics is a rapidly growing field of research based on plasmonic nanostructures. To exploit the full potential of this fascinating class of materials, it is indispensable to tune and optimize the properties of these structures, which requires precise knowledge and optimization of their synthesis processes. Plasmonic silver nanocubes for applications in nonpolar media are obtained by an AgCl-mediated hot-injection method. In this process, catalysis by Fe species is of central importance, as the Fe species influence the reaction in multiple ways, enabling a finely balanced control of the nanocube synthesis. Using electron microscopy, optical spectroscopy, and X-ray photoelectron spectroscopy, it is shown that the Fe species not only direct the reaction of the Ag precursor to the formation of AgCl nanoparticles instead of icosahedral Ag nanoparticles but also enhance the reduction rate of AgCl, from which the Ag nanocubes are formed and grow. Based on these results, a detailed reaction mechanism is proposed. An additional comparison of the effects of different metal ions on the reaction shows that iron ions are highly likely to be specific as catalysts for this synthesis. The results also indicate that the Fe ions are likely present in the form of an organic iron complex, catalyzing the chloride transfer.

## Graphical abstract

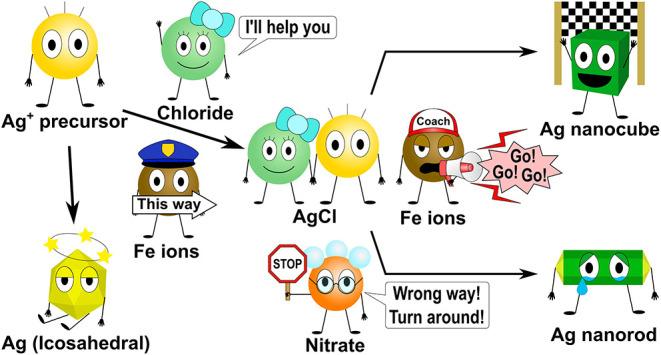

## Introduction

The unique properties of anisotropic silver nanoparticles enable their use in a variety of applications. Their optical and electronic properties, such as the tunable localized surface plasmon resonance (LSPR), can be utilized in electronic devices,^1–3^ sensors,^4–6^ or surface-enhanced Raman spectroscopy (SERS).^7–9^ The shape of the nanoparticles significantly influences their LSPR and the local enhancement of the electromagnetic field. Moreover, the shape and the associated crystal facets can enhance the effect of Ag nanoparticles as antimicrobial agents or in (photo)­catalysis. For example, shapes with sharp edges enhance antimicrobial effectiveness,^10^ and the {111} facet of Ag nanoparticles promotes oxygen reduction reactions or the reduction of methylene blue,^11,12^ while the {100} facet improves styrene oxidation and ethylene epoxidation.^13,14^

One popular shape is the nanocube. The ideal nanocube has sharp corners, which can amplify electromagnetic hotspots that increase SERS activity,^15^ and consists exclusively of {100} facets, which is advantageous for several catalysis applications.^13,14^ However, growing an Ag nanocube is challenging. Since a nanocube is a single-crystal, it must grow from a single-crystalline (SC) seed nanoparticle. Usually, however, multiply twinned (MT) seed nanoparticles are formed exhibiting predominantly or entirely {111} facets, as this is the most thermodynamically stable crystal facet of Ag.^16,17^ Thus, the first challenge is to obtain only Ag (SC) nanoparticles by either preventing the formation of Ag (MT) nanoparticles or oxidatively etching them away. An Ag (SC) seed nanoparticle has a cubic crystal structure with 14 crystal facets: 6 {100} facets and 8 {111} facets. To grow a nanocube, the second challenge is to stabilize the thermodynamically less stable {100} facets, allowing only the {111} facets to grow. Ultimately, this growth results in the formation of a nanocube.

To date, several strategies have been developed for synthesizing Ag nanocubes. The Xia group designed and extensively studied protocols for synthesizing polar Ag nanocubes. Over the past two decades, this group has optimized the polyol approach, which, in general, uses AgNO_3_ as Ag precursor, poly­(vinylpyrrolidone) (PVP) as stabilizing and shape-directing agent, and ethylene glycol as solvent and reducing agent.^18^ The addition of chloride in combination with oxygen improves the oxidation of the Ag (MT) nanoparticles,^19^ an Fe­(II)/Fe­(III) redox system increases the edge sharpness of the nanocubes by scavenging excessive oxygen,^17^ and proton, hydrogen, and sulfide sources help balance the reduction and oxidation rate.^20–23^ With other polyol solvents, different Ag precursors, or additional stabilizing agents, the size of the nanocubes can be adjusted, the controllability of the synthesis can be increased, and the sharpness of small nanocubes can be improved.^24–26^ After the first synthesis of Ag nanocubes by the polyol method was reported, further protocols were developed. Other polar nanocubes were obtained using seed-mediated and aqueous approaches.^27–30^

A substantial number of applications also exist for nonpolar Ag nanocubes. These applications include catalysis in nonpolar media, self-assembly at interfaces, or the use as part of flexible plasmonic substrates due to their long-chain organic ligands.^31,32^ However, there are limited reports on nonpolar syntheses of Ag nanocubes, with three main approaches. Ma et al. used Fe­(III) species as an oxidizing agent in a heat-up process with isoamyl ether.^33^ The obtained nanocubes were small (∼13.5 nm) but possessed truncated corners. Polaravapu et al. and Pan et al. synthesized silver nanocubes in dichlorobenzene as a solvent.^34,35^ Although the resulting nanocubes have sharp edges, the synthesis is time-consuming, requiring a duration of 48 h. Additionally, dichlorobenzene is a CMR substance that must be handled with care. Peng and Sun, as well as our group, developed a protocol for preparing silver nanocubes via the formation of AgCl.^36,37^ During this hot-injection approach, an Ag precursor reacts with a chloride precursor in a mixture of oleylamine (OlAm) and dioctyl ether or dibenzyl ether to form AgCl and Ag (MT) nanoparticles. The AgCl, in turn, is reduced by OlAm to Ag (SC) nanoparticles, which grow into nanocubes while the Ag (MT) nanoparticles are oxidized. We could show that a trace amount of Fe species is essential for the successful growth of nanocubes.^37^ However, the exact influence on the formation mechanism remains unclear.

Iron and other metallic species are known for their catalytic properties in chemical synthesis and are also used in several protocols for silver nanoparticle preparation. The Xia group used an Fe­(II)/Fe­(III) redox system to control the etching of Ag nanoparticles in their polyol synthesis.^17^ Fe­(II) scavenges excess oxygen so that the Ag (MT) nanoparticles are etched, but the sharp edges of the nanocubes are preserved. The resulting Fe­(III) is reduced again by the solvent ethylene glycol. They also used an Fe­(II)/Fe­(III) redox system in their nonpolar synthesis.^33^ While no nanocubes were obtained without iron, the percentage increased to 55% with a trace amount of Fe­(acac)_3_, corresponding to only a thousandth of the silver content, and to 70% with the same amount of FeCl_3_. The authors concluded that the defect-rich Ag (MT) nanoparticles are oxidized by Fe­(III), which is then restored by oxygen from the air. In addition, the etching power increases in the presence of chloride. An example of a catalyzed reduction of Ag­(I) to Ag(0) is given in the work of Li et al.^38^ They reduced the reaction time and enhanced the uniformity of the resulting Ag nanoparticles significantly by introducing Fe­(NO_3_)_3_ into a heat-up synthesis with AgNO_3_ in an oleic acid/OlAm solvent mixture. Their proposed mechanism involves the reduction of Fe­(III) by OlAm and the reduction of Ag­(I) by Fe­(II) and OlAm.

The present study focuses on identifying the role of the Fe species during the synthesis of Ag nanocubes via AgCl in a nonpolar solvent. First, the influence of different Fe ion concentrations on the obtained Ag nanocubes after 1 h and on the initial Ag seed and AgCl formation is examined by SEM/TEM, XRD, and UV/vis measurements. A detailed reaction mechanism is proposed, in which the Fe species are involved at two different stages: (1) They shift the ratio between the initially formed Ag (MT) nanoparticles and the AgCl nanoparticles significantly in the direction of the latter by enhancing the formation of AgCl nanoparticles. (2) They increase the reduction rate of the AgCl nanoparticles, leading to a faster overall reaction. However, since the oxidation rate seems not to be affected by the Fe species, a larger part of the Ag (MT) nanoparticles grows into nanorods before they can be etched when the concentration of Fe species is increased. However, if the first and second effects are balanced, a high yield of nanocubes can be obtained in a relatively short time. AAS, ICP-OES, and XPS measurements support the considerations toward the reaction mechanism and suggest an organic iron complex as catalyst. Furthermore, the introduction of different metal ions instead of Fe­(III) ions shows that Fe ions specifically catalyze the synthesis, leading to the assumption that an organic iron complex acts as a chloride transfer agent.

## Results and Discussion

In a previous study, a controlled and practicable synthesis of silver nanocubes in nonpolar solvents was developed using iron ions as catalysts to shorten the reaction time and enhance cube formation.^37^ The aim of the current study is to examine the complex influence of this catalyst in more detail.

In brief, the synthesis proceeds as follows: An Ag precursor consisting of AgNO_3_ (0.6 mmol) and oleylamine (OlAm, 2.97 mL) is injected into a Cl precursor solution, which contains oleylammonium chloride (OlAmoCl, 0.525 mmol), dibenzyl ether (DBE, 20 mL), oleylamine (OlAm, 2.6 mL), and a trace amount of FeCl_3_ (1.1 μmol) at 260 °C. After the injection, AgCl and multiply twinned (MT) Ag nanoparticles are formed simultaneously. While OlAm reduces the AgCl to single crystalline (SC) Ag nanoparticles, the Ag (MT) nanoparticles are oxidized by nitrate (from the AgNO_3_ precursor and eventually additionally added nitrate, *e*.*g*., ammonium nitrate^37^), which is supported by chloride. The Ag (SC) nanoparticles, therefore, grow at the expense of the Ag (MT) nanoparticles, and since the chloride also acts as shape-directing agent, this process ultimately leads to the growth of Ag nanocubes. If the oxidation is insufficient, the product received at the end of the synthesis contains a fraction of Ag (MT) nanoparticles that have grown into nanorods. However, if the reduction rate is slowed down, *e*.*g*., by shifting the equilibrium toward AgCl by increasing the chloride concentration, the product obtained still contains AgCl.

### Influence of Fe Species on the Reaction Product

At first, the influence of the iron catalyst on the final reaction product was examined by varying the amount of Fe ions while keeping the amount of chloride fixed.

The properties examined were the edge length of the nanocubes, the number percentage of Ag (MT) nanoparticles, the molar percentage of AgCl, and the duration of the initial reaction. The initial reaction is accompanied by a color change in the reaction solution: AgCl formed after injection of the Ag precursor appears milky white with a yellowish coloration due to the Ag seeds. As the AgCl is reduced to Ag and the Ag nanoparticles grow, the color changes to a darker yellow and orange until finally a greenish-gray mixture is obtained when the Ag (SC) nanoparticles begin to grow into a cubic shape. The further reaction includes the final formation of the cubic shape and the general growth of the nanoparticles. The results are displayed in Figure 1a along with SEM images of two exemplary experiments (Figure 1b,c).

**Figure 1:**
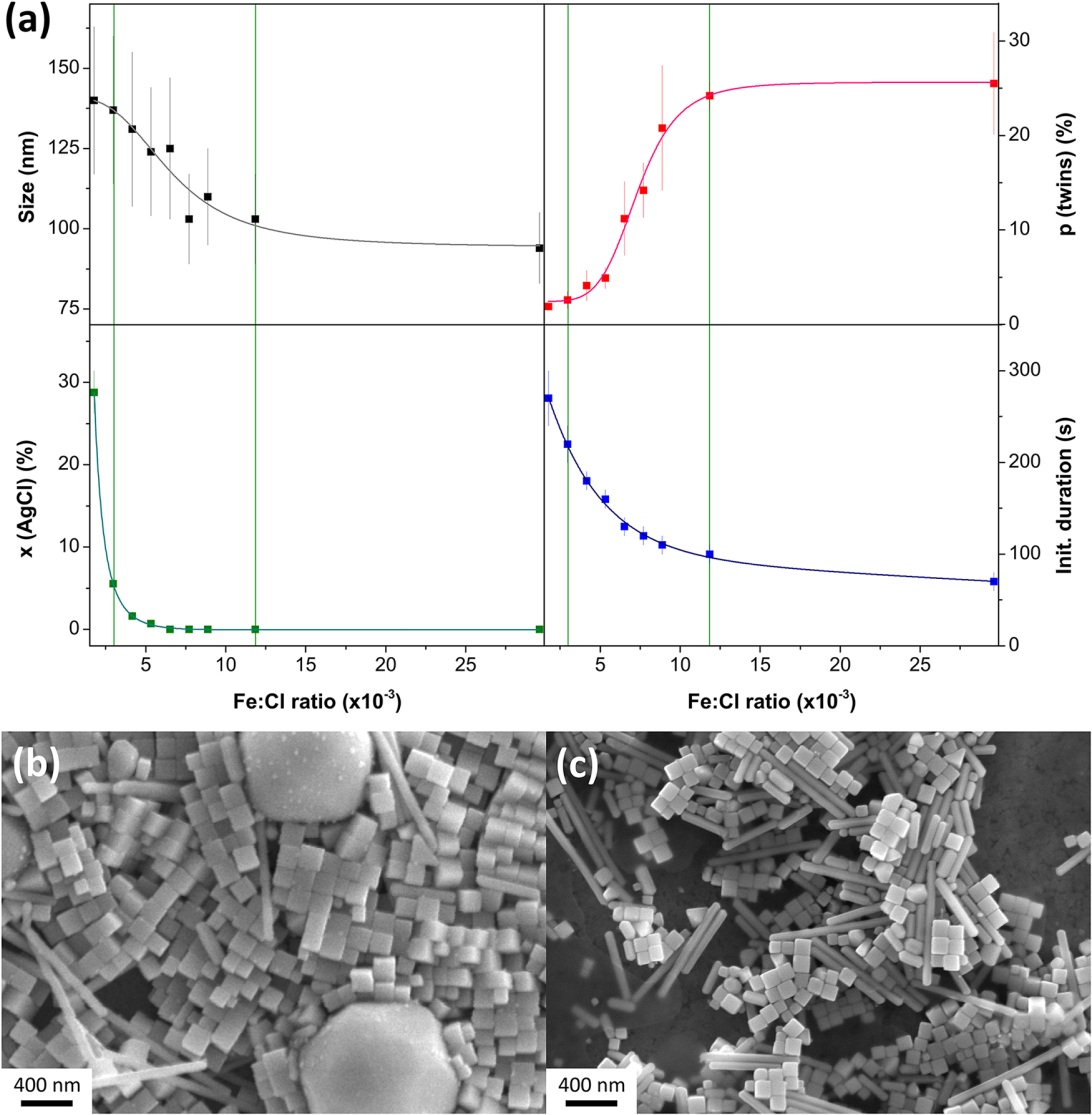
Study on the influence of the Fe species content on the reaction product and speed: (a) shows the edge length of the nanocubes, the number percentage of Ag (MT) nanoparticles (p (twins)), and the molar percentage of AgCl (x (AgCl)) in the product obtained after 1 h reaction time as well as the duration of the initial color change (from injection to constant color) in dependence of the Fe:Cl ratio. Figures (b) and (c) show SEM images of the products obtained with Fe:Cl ratio of 3.0 × 10^–3^ and 11.8 × 10^–3^ (corresponding to 1.9 and 7.4 μmol Fe species), respectively, as indicated by the green vertical lines in Figure (a).

If the Fe:Cl ratio is low, the initial color change takes up to 5 min, and the reaction mixture retains a milky white portion in its color. Accordingly, up to almost 30% AgCl can be found in the final product. The Ag nanocubes obtained are relatively large, with an edge length of about 140 nm, and have an increased polydispersity of over 15%, compared to the nanocubes from the standard synthesis with an edge length of ∼ 100 nm and a polydispersity of less than 10%. However, the percentage of Ag (MT) nanoparticles, recognizable as rods in the final product,^39^ is low at 2%. Figure 1b shows an SEM image of a sample with a low Fe:Cl ratio (3.0 × 10^–3^). The majority of the particles are Ag nanocubes with some rods interspersed between them. There are also two large, spherical particles that can be identified as AgCl since they decompose under electron irradiation in the SEM. This is a well-known behavior of AgCl nano- and microparticles.^40^

When the Fe:Cl ratio is increased, all examined properties undergo significant changes. While the edge length of the nanocubes decreases, the percentage of the Ag (MT) nanoparticles increases. However, both seem to reach a threshold value of approximately 100 nm and 25%, respectively, at an Fe:Cl ratio of 11.8 × 10^–3^ (7.4 μmol Fe species). The polydispersity of the Ag nanocubes also decreases to around 10% at this ratio. The proportion of AgCl in the final product diminishes even faster. Above an Fe:Cl ratio of ∼ 6.0 × 10^–3^ (3.8 μmol Fe species), neither Cl could be found in EDX nor AgCl could be detected in XRD. Although the other properties seem to change asymptotically with an increasing Fe:Cl ratio, the duration of the initial color change appears to decay exponentially. An SEM image of a sample with a high Fe:Cl ratio (11.8 × 10^–3^) is shown in Figure 1c. In comparison with the image in Figure 1b, no large AgCl particles can be seen. However, the image shows significantly more rods next to the Ag nanocubes. The XRD data presented in Figure S1 in the Supporting Information corroborate the findings regarding AgCl. AgCl diffraction peaks are visible in the three samples with the lowest Fe ion concentration, albeit barely at an Fe:Cl ratio of 4.1 × 10^–3^. In contrast to the peak intensity of the pattern of bulk Ag (COD 9008459), the (200) diffraction peaks found in the presented diffractograms are more pronounced than the (111) diffraction peaks. That is because Ag nanocubes are terminated by {100} planes and usually assemble horizontally during sample preparation.^41^

The obtained reaction data can mainly be explained by an enhanced reduction of Ag^+^ to Ag^0^ in the presence of Fe species. If the amount of Fe species is low and the reduction is, therefore, slower, the AgCl cannot be completely reduced in 1 h reaction time. Accordingly, the nucleation of Ag (SC) nanoparticles is delayed, resulting in fewer nuclei being formed. This would result in fewer, larger nanocubes with a broader size distribution. When the concentration of Fe species increases and the reduction rate accelerates, AgCl is reduced faster, which leads to a faster initial color change and a faster overall reaction. The AgCl can be reduced completely in the given reaction time. More Ag (SC) nuclei are formed in a shorter time, and the edge length of the nanocubes becomes smaller with a narrower size distribution. If the reduction rate is increased, the growth rate of the silver nanoparticles also increases, as monomers are formed faster. This also explains an increase in the Ag (MT) nanoparticles, as some Ag (MT) nanoparticles grow faster than they can be oxidized. This, in turn, would lead to a decreasing edge length of the Ag nanocubes, as fewer monomers are available for the growth of the nanocubes.

However, an enhanced reduction rate does not explain why the edge length of the Ag nanocubes and the percentage of the MT nanoparticles asymptotically approach a threshold value. This can only be the case if the number of nuclei is limited when the concentration of iron ions exceeds a certain value. Therefore, the initial phase of the reaction is studied in the following section.

### Influence of Fe Ions on the Early Stage of the Reaction

To study the different reaction behaviors during the early stage depending on the iron ion concentration, syntheses with 1.1 μmol Fe­(III), with 2.6 μmol Fe­(III), and without Fe­(III) ions were examined. Six samples were drawn over the first 2 min for each series. Please note that the addition of different trace amounts of FeCl_3_ to the reaction only insignificantly changes the Ag:Cl ratio: Without the addition of FeCl_3_, the Ag:Cl ratio is 1.143, while it changes to 1.124 when 2.6 μmol FeCl_3_ is added, corresponding to a difference of 1.6%.

The formation of Ag nanoparticles as well as the formation and dissolution of AgCl was examined via SEM and UV/vis spectroscopy since the reaction progress can already be followed by an initial color change from white to yellowish and orange-red to greenish-gray (s. Figures S2 and S3, Supporting Information). However, the solvent mixture absorbs in the UV region below 350 nm, which is why AgCl could not be examined via UV/vis spectroscopy, as its absorption is also in the UV region.^42^

Figure 2 shows the absorbance spectra of the samples with 1.1 μmol Fe­(III), as well as the size distributions of the AgCl particles found. As shown in Figure 2a,b, the absorbance spectra exhibit two peaks at 404 and 418 nm, which increase in intensity during the first 30 s of the reaction. Both peaks can only originate from small Ag nanoparticles and are found in a region where the absorption of small Ag nanoparticles would be expected.^43–45^ While the absorbance after 2 s is almost not visible, some Ag nanoparticles around 5–8 nm in size could be found in the SEM (s. Figure S4, Supporting Information). After 5 s, the absorbance peak at 418 nm is mainly visible with a small shoulder (which resembles a tailing) toward lower wavelengths. In addition to the 5–8 nm Ag nanoparticles, Ag nanoparticles of around 12–14 nm diameter were found. Thus, the smaller nanoparticles can be attributed to the peak at 418 nm, and the larger nanoparticles can be attributed to the peak at 404 nm. After 30 s, the ratio of the two absorbance peaks has shifted, and the peak at 418 nm appears as a shoulder of the peak at 404 nm.

**Figure 2:**
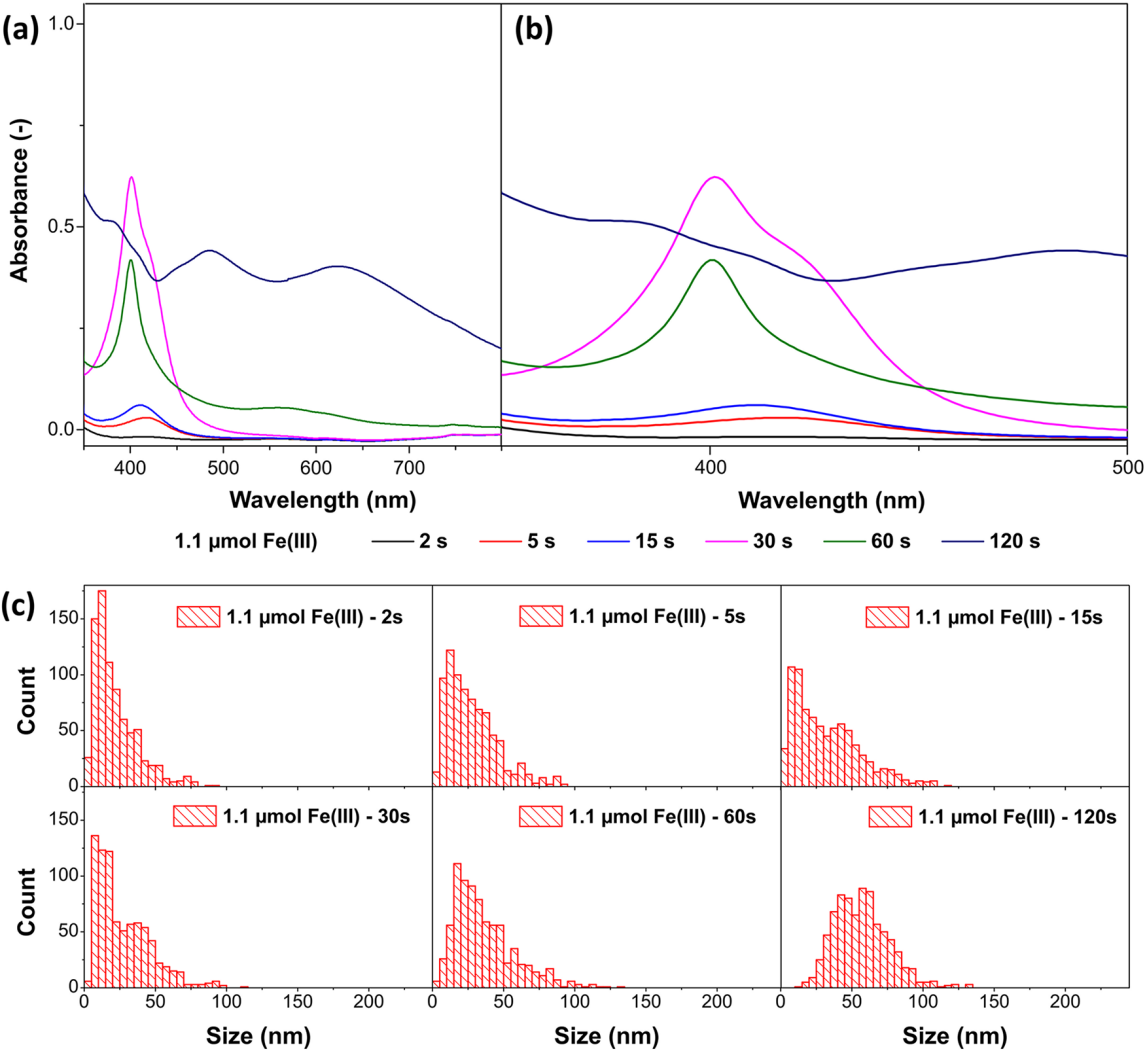
Time-dependent analysis of the early stage of the Ag nanocube synthesis with the addition of 1.1 μmol Fe­(III) showing (a) the UV/vis absorbance spectra over the whole measured range from 350 to 800 nm, (b) the UV/vis absorption peaks of the Ag nanoparticles in detail from 350 to 500 nm, and (c) the size (diameter) distribution of the AgCl nanoparticles obtained from SEM after 2, 5, 15, 30, 60, and 120 s.

Since both absorbance peaks are distinct and do not shift continuously over the observed duration, and since the absorbance maximum of the larger Ag nanoparticles does not correlate with the absorbance maximum of OlAm-capped Ag nanoparticles of the same size in previous studies,^43^ it is unlikely that the different sizes of the Ag nanoparticles cause the shift. The most probable cause is the presence of different ligands or different materials surrounding the Ag nanoparticles. Ligands influence the electronic structure of nanoparticles because the electron conductivity in the outer layer of the particle, which is chemically bound to the ligands or the outer shell material, is lower than in the core. The extent of this effect depends on the chemical nature of the bound element and the chain length of the ligand molecules.^45^ This leads to the assumption that the Ag nanoparticles with an absorbance maximum at 418 nm are Ag nanoparticles that form upon thermal decomposition of the Ag-OlAm precursor (which in the following will be referred to as primary Ag nanoparticles) and are assumed to be coated with OlAm ligands.^43^ The Ag nanoparticles with an absorbance maximum at 404 nm are Ag nanoparticles that have evolved from AgCl nanoparticles (as proposed by Peng et al.,^46^ and which in the following will be referred to as secondary Ag nanoparticles) and might be passivated by an AgCl layer.^14^ The formation of an AgCl monolayer and possibly the absence or exchange of long-chained ligands cause the absorbance maximum to blue-shift.^45^ An XPS analysis of the final Ag nanocubes (more details in the following section) confirms the presence of AgCl and the absence of nitrogen, supporting the aforementioned theory.

In contrast, the possibility of loss of OlAm ligands due to passivation by AgCl of already existing silver nanoparticles can be ruled out, as the primary particle morphology from thermal decomposition is the icosahedral shape.^47^ However, only nanocubes and nanorods can be found in the final product, but no icosahedral morphologies (s. Figure 1c). This means that the primary Ag nanoparticles are completely removed by etching and, hence, do not lose their ligands due to passivation, as well as that secondary Ag nanoparticles can also be multiply twinned. Since the etching is assumed to be a continuous process and the intensity of the peak at 418 nm significantly decreases after 30 s, primary Ag nanoparticles appear to form in the first ∼30 s. The intensity of the peak at 404 nm also decreases after 30 s, but this is due to the incipient growth of these Ag nanoparticles into nanocubes. As the size of the secondary Ag nanoparticles increases, the absorption spectrum is more strongly influenced by scattering and begins to show plasmon resonances that correlate with those of a nanocube, which can be clearly seen in the spectrum of the sample at 120 s.^48^

The formation of the AgCl nanoparticles can be followed by their size distributions shown in Figure 2c. It seems that AgCl forms rapidly after the injection of the Ag-OlAm precursor into the hot Cl-precursor solution, as the particle size is already distributed to sizes over 50 nm after 2 s. However, the main size fraction is below 20 nm. Over the course of 30 s, the distribution does not change significantly. It tends slightly toward larger sizes, although the main size fraction is still below 20 nm. This indicates that the precursor still forms new AgCl nanoparticles, especially when considering that small AgCl nanoparticles are transformed into secondary Ag nanoparticles.^46^ Since primary Ag and AgCl nanoparticles form for the first ∼30 s, it can be assumed that the Ag precursor needs about this time to react completely. After this time, the central part of the size distribution of the AgCl nanoparticles shifts to sizes above 20 nm and takes on a Gaussian shape. Simultaneously, the secondary Ag nanoparticles grow, and particles with sizes around 12–14 nm are no longer found. This indicates that only the small AgCl nanoparticles (below 20 nm) transform into Ag nanoparticles, while the larger AgCl nanoparticles enable the growth of the Ag nanoparticles during their reduction. No indication of multiple secondary Ag nanoparticles nucleating on one large AgCl nanoparticle, as suggested for similar syntheses,^49,50^ could be found during the SEM examinations (s. Figure S4, Supporting Information).

The presented results as well as previous studies, lead to the following theory:^36,46^ After its injection, the Ag-OlAm precursor is continuously thermally decomposed and forms primary, mainly MT-Ag nanoparticles, which are then oxidized by nitrate, which is supported by chloride. At the same time, the Ag precursor reacts with OlAmoCl and forms AgCl nanoparticles, initially at a rapid rate, then more slowly. Shortly after the formation of AgCl nanoparticles, secondary, mainly SC-Ag nanoparticles transform from small AgCl nanoparticles. The Ag precursor reacts for about 30 s, while the oxidation of the primary Ag nanoparticles and the formation of the secondary Ag nanoparticles takes slightly longer (approximately 5–10 s) until no primary Ag nanoparticles and small AgCl nanoparticles are left. The secondary Ag nanoparticles grow as a result of the reduction of the large AgCl nanoparticles.

When the concentration of Fe­(III) is increased, the results of the previous section suggest a faster Ag nanocube formation, but with a larger proportion of Ag nanorods present. The results of the examination of the early stage of synthesis with the addition of 2.6 μmol Fe­(III) are presented in Figure 3. Comparing the absorbance data in Figure 3a,b with the absorbance data in Figure 2a,b, *i*.*e*., with the synthesis with less Fe­(III), the absorbance peak of the primary Ag nanoparticles (418 nm) is significantly lower, which means that fewer primary Ag nanoparticles are present. The reason could either be an enhanced oxidation of these nanoparticles or their inhibited formation. The fact that at the end of the synthesis, the more nanorods (Ag (MT) particles) are present, the more Fe­(III) is used, indicates that the latter is the case. A second indication is that the Ag precursor seems to be consumed faster if more Fe­(III) is added to the synthesis. The UV–vis absorbance data and the size distribution of the AgCl nanoparticles suggest that primary Ag and AgCl nanoparticles are formed within the first approximately 15 s instead of the first approximately 30 s with 1.1 μmol Fe­(III). This also leads to the assumption that Fe­(III), or Fe ions in general, catalyzes the formation of AgCl.

**Figure 3:**
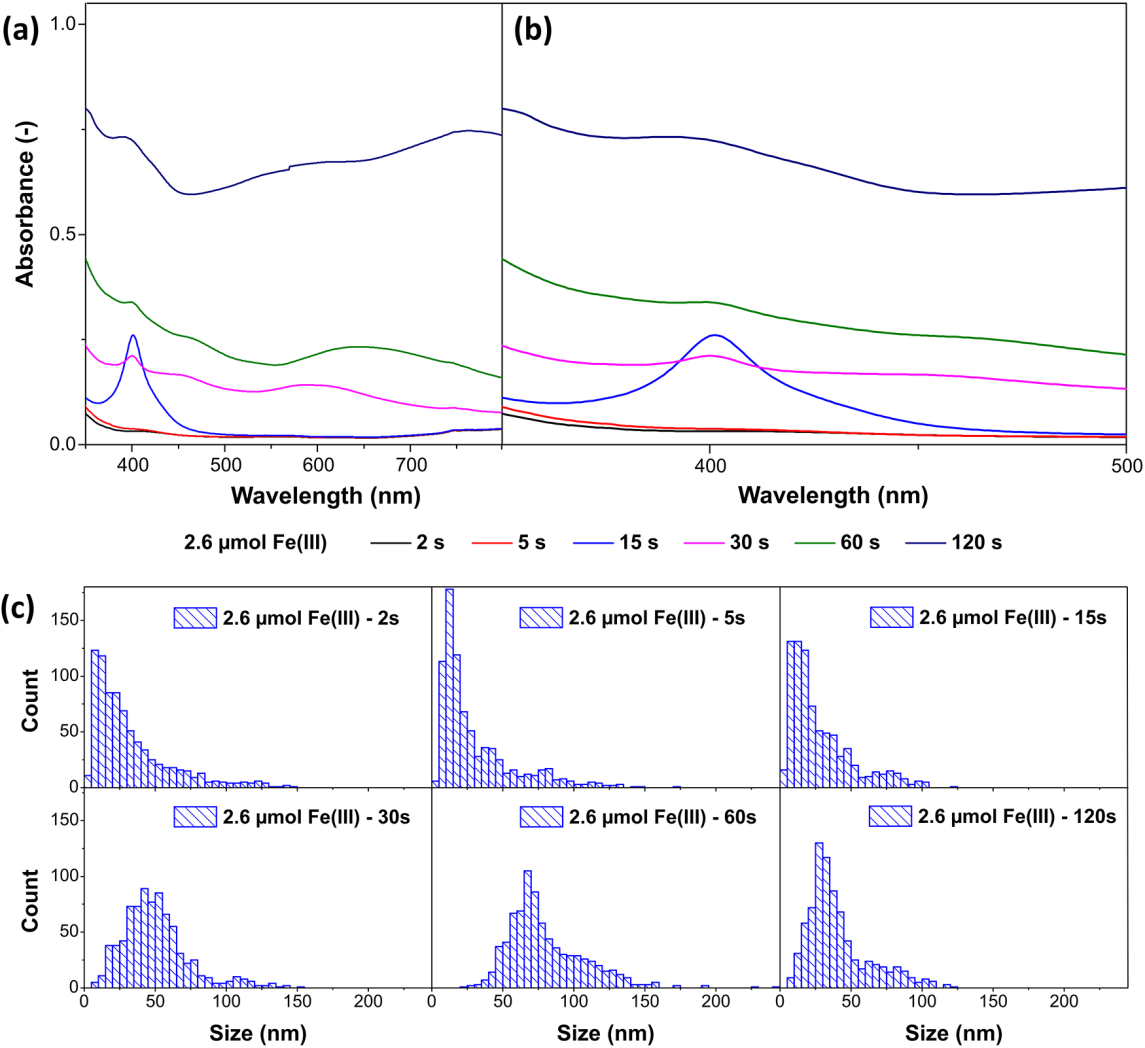
Time-dependent analysis of the early stage of the Ag nanocube synthesis with the addition of 2.6 μmol Fe­(III) showing (a) the UV/vis absorbance spectra over the whole measured range from 350 to 800 nm, (b) the UV/vis absorption peaks of the Ag nanoparticles in detail from 350 to 500 nm, and (c) the size (diameter) distribution of the AgCl nanoparticles obtained from SEM after 2, 5, 15, 30, 60, and 120 s.

The presented data (Figure 3 and Figure S5, Supporting Information) also suggest that the reduction of AgCl is enhanced. Compared to the results from the experiments with 1.1 μmol Fe­(III), the secondary Ag nanoparticles grow larger and take on a cubic shape in a shorter time, as indicated by the development of the plasmon resonance absorption. Accordingly, the size distribution of the AgCl nanoparticles shifts back to smaller sizes at 120 s, as the AgCl particles are reduced more quickly. This means that Fe­(III), or Fe ions in general, also catalyze the reduction of AgCl, as already suggested in the previous section.

Considering that the reduction of AgCl is enhanced, but the oxidation of Ag (MT) nanoparticles is not, the increase in Ag nanorods can be explained. Studies show that Ag nanocubes, as well as nanowires, can be obtained via heterogeneous nucleation on AgCl, depending on the chemical environment.^49,50^ Therefore, it is likely that this process produces a statistically distributed mixture of secondary Ag (SC) and Ag (MT) nuclei. Supposing that the etching of Ag (MT) nuclei does not depend on the Fe­(III) concentration, it can be assumed that the etching process is the same for syntheses with the same nitrate and chloride concentrations. Thus, a larger number of Ag (MT) nuclei can grow before they are etched if the reduction rate of AgCl increases at higher Fe­(III) concentrations. This means that more Ag nanorods are present at the end of a synthesis if the amount of Fe­(III) is higher. It also explains the thresholds for the edge length of the Ag nanocubes and the percentage of nanorods when a certain concentration of Fe­(III) is reached (s. Figure 1a). When a certain amount of Fe­(III) has increased the reduction rate of AgCl to a point where all secondary Ag (MT) nuclei can grow before they are etched, the final ratio of Ag nanocubes to nanorods can no longer change. Accordingly, the number of particles does not change further, and their size remains constant.

A third series of experiments was performed without the addition of Fe­(III). Over the entire examined period, the absorbance data shown in Figure 4a,b indicate a steadily increasing intensity, which is significantly higher than that observed in the experiments with the addition of Fe­(III). Also, AgCl nanoparticles appear to form only during a short period after the start of the reaction, as indicated by the histograms in Figure 4c. The main AgCl particle fraction shifts toward larger sizes already after a reaction time of 5 s. The formation of new primary Ag nanoparticles continues for more than 120 s, while new AgCl nanoparticles are only formed within the first approximately 2 s. This is supported by the comparison of the initial color change of a reaction with Fe­(III) ions and without Fe­(III) ions added (s. Figures S2 and S3, Supporting Information). It is evident that the color of the reaction solution with added Fe­(III) ions remains white for longer than 60 s, while the color of the solution without added Fe­(III) ions only exhibits a faint white color for about 2 s and then changes to a yellow color that becomes increasingly intense. These results confirm the theory that Fe ions catalyze the reaction of the precursor with OlAmoCl. Without the addition of Fe­(III), both the consumption rate of the Ag precursor as well as the formation rate of AgCl are significantly lower. However, the average size of the AgCl nanoparticles increases over time, indicating that a reaction between the Ag precursor and the OlAmoCl takes place. Alternatively, the growth could also be due to the oxidation of the primary Ag nanoparticles. Furthermore, the slow growth of the Ag nanoparticles (s. Figure S6, Supporting Information) confirms the reduced reduction rate of AgCl due to the absence of Fe ions. The growth of the Ag nanoparticles at this stage is mainly due to the thermal decomposition of the Ag precursor.

**Figure 4:**
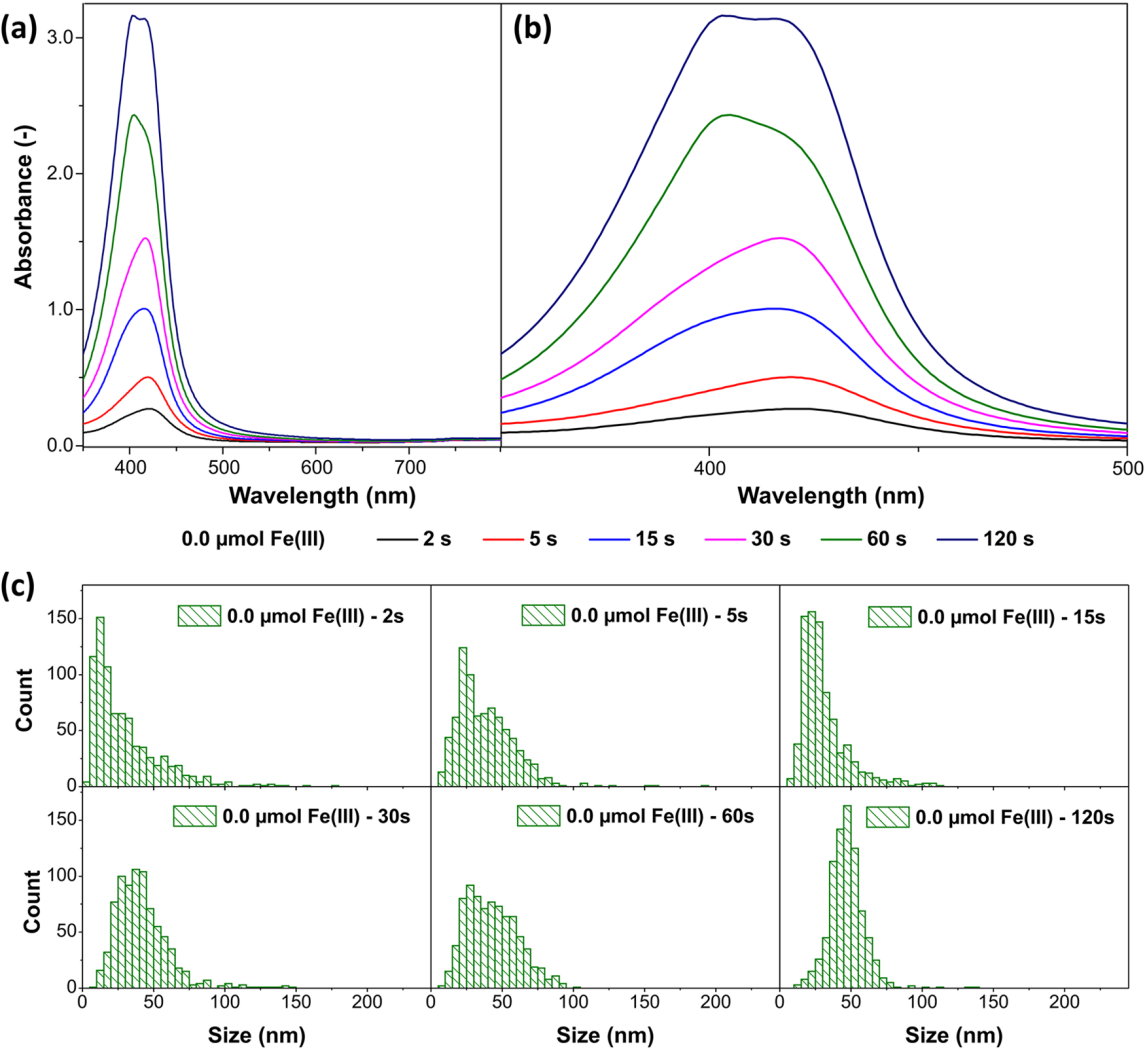
Time-dependent analysis of the early stage of the Ag nanocube synthesis without the addition of Fe­(III) showing (a) the UV/vis absorbance spectra over the whole measured range from 350 to 800 nm, (b) the UV/vis absorption peaks of the Ag nanoparticles in detail from 350 to 500 nm, and (c) the size (diameter) distribution of the AgCl nanoparticles obtained from SEM after 2, 5, 15, 30, 60, and 120 s.

The absorbance spectra also show the two peaks assigned to the OlAm-capped primary Ag nanoparticles (418 nm) and the chloride-capped secondary Ag nanoparticles (404 nm). Both peaks grow steadily over time. However, without small AgCl nanoparticles and a drastically reduced reduction rate of AgCl due to the lack of a catalyst, the amount of secondary Ag nanoparticles should be low, and their formation, if it occurs at all, should stop early. Therefore, it can be assumed that the OlAm ligands of the primary Ag (MT) nanoparticles detach due to passivation of the nanoparticles by AgCl or a ligand-change in this case. This is supported by the composition of the Ag nanoparticles after 1 h reaction time, which was determined by analyzing TEM images. The sample consists of ∼80% Ag (MT) nanoparticles and ∼20% Ag (SC) nanoparticles, which corresponds to the composition of Ag nanoparticles obtained from a synthesis in dodecane by reduction of a precursor with fatty amines at 170 °C.^44^ A higher amount of Ag (SC) nanoparticles would be expected if secondary Ag nanoparticles are formed.

Scheme 1 summarizes the previous conclusions and illustrates the different reaction stages and pathways as well as the various influences of the reaction agents.

**Scheme 1:**
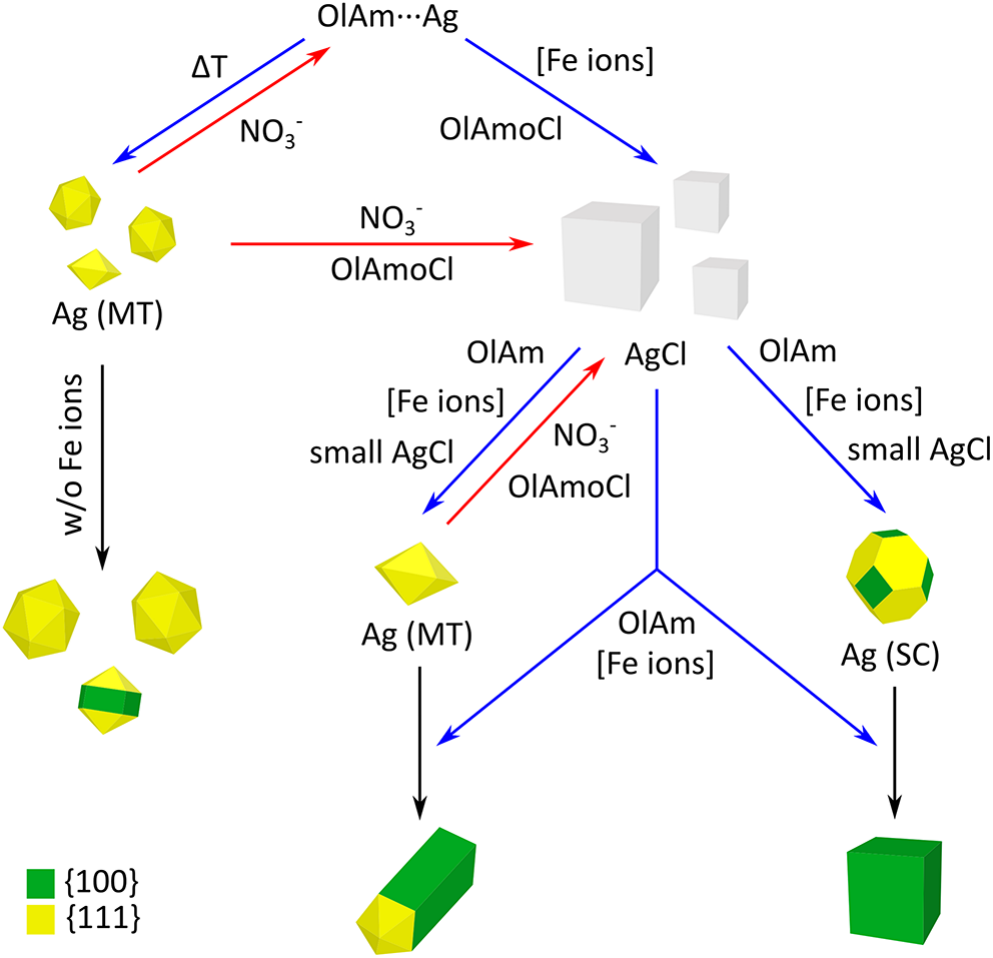
Schematic Illustration of the Mechanisms and influences that Lead from the Precursor to the Final Products^a^

a The Ag-oleylamine (OlAm) precursor can undergo two different pathways: (1) It thermally decomposes into primary, multiply twinned (MT) Ag nanoparticles, which can be oxidized again by nitrate. (2) It reacts with oleylammonium chloride (OlAmoCl) to form AgCl, which is catalyzed by Fe ions. If there are no Fe ions present, the formation rate of primary Ag (MT) nanoparticles is higher than the oxidation rate, so that they grow and are part of the final product. If Fe ions are present, the reduction of the initially formed AgCl nanoparticles by OlAm is also catalyzed. Small AgCl nanoparticles can either transform into secondary Ag nanoparticles (MT), which are oxidized again, or into single-crystalline (SC) nanoparticles. The Ag (MT) nanoparticles grow into nanorods, while the Ag (SC) nanoparticles grow into nanocubes when the large AgCl nanoparticles are reduced. The blue arrows indicate reduction and the red arrows oxidation, while the black arrows indicate further particle growth.

When the Ag-OlAm precursor is injected into the 260 °C hot chloride precursor solution, two different pathways coincide. First, the precursor decomposes thermally and forms primary Ag (MT) nanoparticles. These are oxidized by nitrate at a constant rate and possibly form an Ag-OlAm complex again or form AgCl with the help of OlAmoCl, since chloride supports the etching process.^37^ Second, AgCl nanoparticles are formed when the precursor reacts with OlAmoCl. This reaction is catalyzed by Fe ions, so that when enough Fe ions are present, mainly AgCl nanoparticles are formed, and the few primary Ag (MT) nanoparticles formed are completely etched away. If too few Fe ions are present, mainly primary Ag (MT) nanoparticles are formed, which cannot be completely etched and can grow into Ag nanorods.

The AgCl nanoparticles are reduced by OlAm, which is also catalyzed by Fe ions. As the decomposition/reaction of the Ag precursor occurs within a few seconds to several minutes (depending on the amount of Fe ions used), both processes take place simultaneously for a period of time. If the AgCl nanoparticles are small (∼20 nm), they transform into secondary Ag nanoparticles during reduction. These can be either Ag (SC) or decahedral Ag (MT) nanoparticles. The secondary Ag (MT) nanoparticles are also etched as long as they have not reached a critical size. Although it is possible that the Ag (SC) nanoparticles are also etched, the oxidation of the less stable Ag (MT) nanoparticles is preferred, which means that the oxidation of the Ag (SC) nanoparticles can be neglected.

The secondary Ag nanoparticles grow due to the reduction of the remaining, mainly large AgCl nanoparticles. Because of the present chloride, they either grow into nanorods (MT) or nanocubes (SC). The higher the Fe ion concentration, the faster the reduction, which means that more Ag (MT) nanoparticles reach the critical size before they are etched. A higher Fe ion concentration thus shifts the ratio of nanorods to nanocubes toward nanorods until the statistical ratio of initially formed Ag (MT) to Ag (SC) is reached, which appears to be about 1:3 (s. Figure 1a).

### Surface and Elemental Analysis of Ag Nanocubes

Although the interaction points of the Fe ions during the synthesis are now identified, the nature of this interaction remains unknown. If a direct interaction occurs, traces of iron should remain on the final nanoparticles. The surface of the Ag nanocubes was investigated with XPS. For the XPS measurement, the purified Ag nanocubes were pressed into indium foil under argon atmosphere (≤0.1 ppm of O_2_) and introduced into the XPS spectrometer under N_2_ atmosphere without contact to air. The resulting survey spectrum is displayed in Figure 5, and a high-resolution spectrum of Ag 3d is shown in Figure S7 in the Supporting Information.

**Figure 5:**
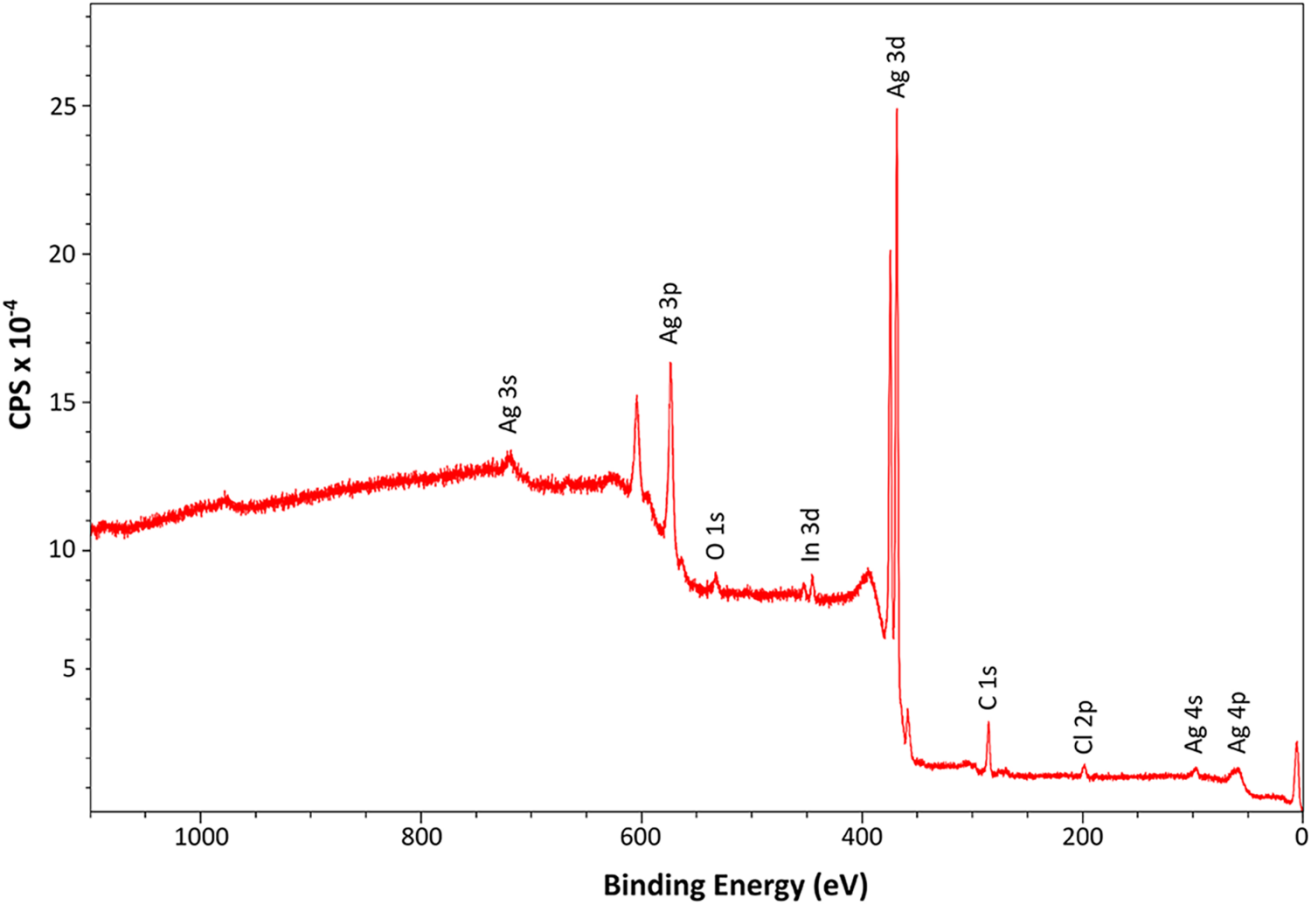
XPS survey spectrum of the purified Ag nanocubes excited by nonmonochromatized Al K_α_ radiation (1486.6 eV). Traces of indium are found since the sample was pressed into indium foil for the measurement.

The survey spectrum is dominated by the intense Ag 3d lines. The electron binding energy of Ag 3d_5/2_ equals 368.2 ± 0.1 eV. Both the binding energy and the small plasmon peaks in the Ag 3d spectrum (s. Figure S7, Supporting Information) are indicative of metallic Ag.^51,52^ The C 1s binding energy (285.0 eV) was used for the correction of surface charging. No iron could be detected in the XPS spectrum, showing that iron does not adsorb onto the silver surface and indicating that the Fe ions have no direct interaction with the silver, such as deposition or alloying, but rather interact in the form of an organic iron complex. The immiscibility of iron and silver also makes the formation of an alloy highly unlikely,^53,54^ which was confirmed by AAS and ICP-OES measurements. A maximum of 50 ppm (related to the amount of substance) iron relative to silver was found, which is significantly lower than the detection limit of the XPS.

Moreover, the XPS spectrum shows a peak for Cl 2p but no indication of nitrogen. OlAm and OlAmoCl, therefore, do not appear to be ligands. The Cl 2p binding energy was determined from the survey spectrum to be 198.1 ± 0.3 eV. A binding energy below 199 eV is a clear indication of inorganic chlorine (chloride), while a binding energy above 200 eV is assigned to organic chlorine.^55^ Moreover, the chloride in AgCl has a binding energy of 197.9 eV, which is within the error margin of the present results.^56^ In correlation with the amount of silver, the chloride concentration can be estimated at ∼10 ± 3 mol %. Based on these results, the assumption of an AgCl monolayer around the Ag nanocubes, which was made in the previous section, can be discussed. Therefore, the following considerations were made: (1) Monolayers cannot be detected using standard XRD measurements, and accordingly, no AgCl could be found using XRD.^57^ (2) The detection limit for SEM-EDX under optimal conditions is approximately 0.1–0.2 wt % (corresponding to 0.3–0.6 mol % in a matrix containing Ag and Cl). The Cl concentration of samples, in which Cl was detected, was typically >1 mol % under the conditions used in this study. Since no Cl was found in the final nanocube sample, this suggests that its content is <1 mol %. (3) The concentration of chloride determined using XPS refers to a layer thickness of 3–5 nm (photoelectron escape depth). For an Ag nanocube with an edge length of 100 nm, the average concentration would be 10–15 times lower (0.5–1.1 mol %). Thus, the Cl content of the final nanocubes is between 0.5 and 1 mol %. (4) An AgCl monolayer around a nanocube with an edge length of 100 nm contains ∼0.5 mol % Cl. This number is consistent with the results found. These findings support the assumption that the secondary Ag nanoparticles are not coated with OlAm ligands but are passivated with an AgCl layer.^14^ This is not contradicted by the position of the Ag 3d_5/2_ signal, as earlier studies could not observe a significant shifting of this signal between metallic silver and AgCl.^58^

However, the nanocubes are colloidally stable, which raises the question of particle stabilization. Based on the XPS results, OlAm as a ligand and chlorine-containing ligands can be ruled out, as only inorganic chlorine and no nitrogen was found. As bare nanoparticles, *i*.*e*., only with an AgCl passivation layer and without ligands, the nanocubes would not be colloidally stable. The only other elements found by XPS are oxygen and carbon, which could indicate oxygen-containing ligands. In an NMR study, Calcabrini et al. reported the oxidation of alkylamines to carboxylic acids by nitrate from a metal nitrate precursor during the synthesis of different metal oxide nanocrystals in a nonpolar medium at high temperatures (300 °C).^59^ The resulting carboxylic acid was detected as a ligand on the nanocrystals, suggesting a similar process is possible in the present system since nitrate and OlAm are present and oxygen and carbon were detected by XPS.

### Effect of Different Metal Ions

So far, the main effects of the Fe ions on the reaction mechanism have been identified, of which one is the enhanced reduction rate of AgCl. An earlier study on the influence of metal ions on the monodispersity of spherical Ag nanoparticles proposes that an electron transfer between Ag­(I) and Fe­(II) is the reason for this, in which Fe­(III) is reduced to Fe­(II) by OlAm. A similar, albeit less pronounced, effect was also observed when Cu­(II) or Cr­(III) ions were added. The effect decreases with the redox potential of the added metal ions.^38^ Thus, to examine the possible reason behind the catalysis in the current study, the results of experiments with different additives were compared: (1) without a metal ion additive, (2) with Fe­(III) ions, (3) with Fe­(II) ions, (4) a different multivalent metal ion with a lower redox potential, Cu­(II), and (5) a metal ion that exists only in one oxidation state, Zn­(II). Figure 6a shows the results of these experiments in terms of the size of the final nanoparticles, the number percentage of Ag (MT) nanoparticles, as well as the molar proportion of remaining AgCl. Since some differences are relatively small, the experiments were repeated three times. The data in Figure 6 are the averaged results of these syntheses. The corresponding SEM/TEM images can be found in Figure 6b–f.

**Figure 6:**
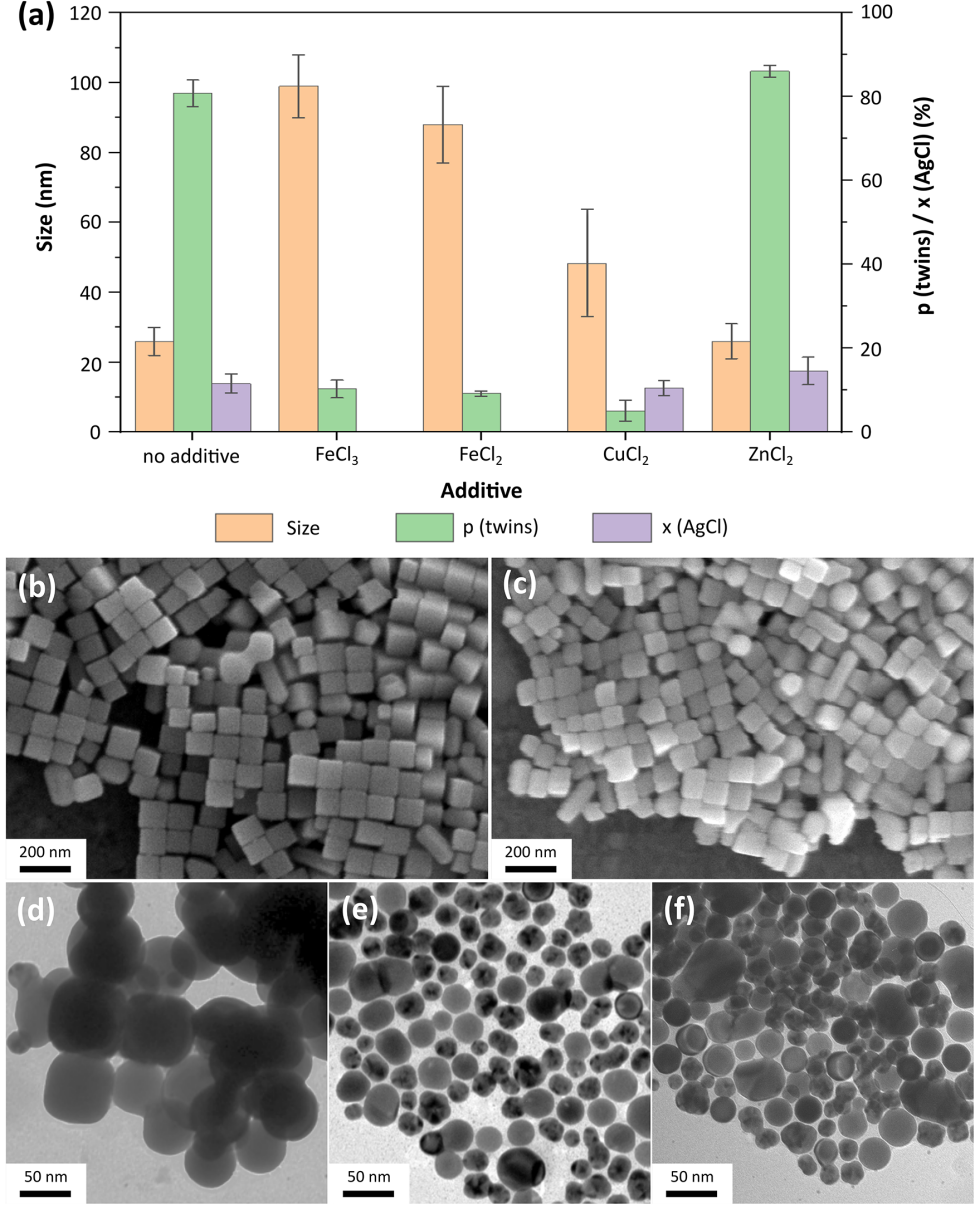
(a) Bar diagram showing the reaction products obtained with different metal ions as additives (1.1 μmol in each case). The size of the obtained silver nanoparticles and the number percentage of MT nanoparticles (p­(twins)), and the mole fraction of AgCl (x (AgCl)) in the sample are compared. No bars for x (AgCl) for FeCl_3_ and FeCl_2_ are displayed since no AgCl was found in these samples. (b–f) SEM/TEM images showing the results of experiments with the addition of 1.1 μmol of different metal ions in the following order: Fe­(III), Fe­(II), Cu­(II), Zn­(II), and no additive.

In the synthesis without additional metal ions, a high number of Ag (MT) nanoparticles (about 80%) is found. Moreover, the Ag (SC) nanoparticles remain relatively small, with a size of 20–30 nm, and do not evolve into nanocubes. Furthermore, the reduction rate is too slow to reduce all the AgCl, and after 1 h of reaction time, slightly more than 10 mol % is still present. The results when Zn­(II) is added to the synthesis are approximately the same. It can, therefore, be assumed that Zn­(II) does not affect the reaction at all.

When the catalytically active Fe­(III) is added to the synthesis, Ag nanocubes are obtained in high yield. Only about 10% Ag (MT) nanoparticles remain in the synthesis, and all AgCl is consumed. The edge length of the nanocubes is 99 ± 9 nm. When Fe­(III) is replaced by Fe­(II), Ag nanocubes are also obtained in high yield with no AgCl remaining. However, with an edge length of 88 ± 11 nm, the nanocubes are slightly smaller and more polydisperse compared to the nanocubes with an Fe­(III) additive. Since the proportion of Ag (MT) nanoparticles and the duration of the initial color change are approximately the same, neither the reduction rate of AgCl nor the oxidation rate of Ag (MT) nanoparticles appear to have changed significantly. The smaller size and higher polydispersity of the Ag nanocubes can thus be explained by assuming that the AgCl formation rate has slowed down; consequently, small AgCl nanoparticles and, subsequently, secondary Ag nanoparticles form over a longer period of time. If the reduction rate of AgCl remains unchanged, more secondary Ag nanoparticles form. In that case, the difference in size between the earlier-formed, already grown Ag nanoparticles and the later-formed Ag nanoparticles will increase. These results indicate that the reaction of the Ag precursor with the chloride precursor to form AgCl is enhanced more strongly by Fe­(III) than by Fe­(II). There could be several reasons for this: (1) Fe­(II) is a weaker catalyst than Fe­(III). (2) The catalysis is based on a redox cycle, which is delayed with Fe­(II) as the initial valence state. (3) Fe­(II) must first be oxidized to Fe­(III) in order to act as a catalyst. This is most likely due to nitrate that is introduced into the reaction mixture together with the Ag precursor.^37^ Since the reduction of AgCl appears not to be affected by the initial valence state, it seems likely that the same Fe species is present at this stage of the synthesis in both experiments. However, the exact reason could not be determined during this study. In any case, the results indicate that the addition of Fe­(III) ions results in slightly less polydisperse nanocubes compared to the addition of Fe­(II) ions.

The use of Cu­(II) results in Ag nanoparticles with a size of 48 ± 13 nm, which are significantly smaller and more polydisperse compared to the nanoparticles obtained with Fe­(III). Moreover, the nanoparticles do not completely evolve into nanocubes, and AgCl remains present after 1 h of reaction time. However, the proportion of Ag (MT) nanoparticles was only half as high as in the presence of Fe­(III). The remaining AgCl indicates that the reduction rate of AgCl is not significantly increased compared to the case without additional metal ions. Since the amount of remaining AgCl and the initial color change are comparable to those in the synthesis without a metal ion additive, increased AgCl formation also seems unlikely. A possible explanation of the effect of Cu­(II) could be an improved oxidation of primary Ag (MT) nanoparticles. While these nanoparticles are etched, the few Ag (SC) nanoparticles formed remain and grow.

The results of this study oppose a mainly redox potential-based effect of the catalyst. When Fe ions are present, the preferred reaction of the Ag precursor is to form AgCl instead of primary Ag nanoparticles. This reaction occurs without a change in the valence state of the Ag^+^ from the precursor or the Cl^–^ from the OlAmoCl, *i*.*e*., without a charge transfer. Moreover, since Cu­(II)→Cu­(I) has a lower redox potential than Fe­(III)→Fe­(II), the reduction of AgCl should be more favored in the case of Cu­(II). An enhanced oxidation in the presence of Cu­(II) seems even more unlikely if a redox potential-based effect is assumed.

To explain the different behavior of the metal ions and the catalysis of the reaction, the formation of a metal–organic complex, possibly consisting of chloride, OlAm, and/or OlAmoCl, is assumed. To estimate and explain the structure and reaction mechanisms of those complexes, the hard and soft acids and bases (HSAB) theory is often used.^60^ Another explanation could be the different solubility of the various complexes, which also leads to different availability of the complexes.^61^

In combination with the previous results, *i*.*e*., only steps involving chloride are catalyzed, and likely no direct Fe–Ag interaction takes place, an organic iron complex acting as a chloride transfer agent could be assumed. The first transfer would be that of the chloride from the OlAmoCl to the Ag^+^ ions to form AgCl at the beginning of the reaction. During the second catalytic step, the reduction of AgCl, the chloride from the AgCl is likely transferred to H^+^ via the Fe catalyst. This leads to the formation of HCl. The H^+^ is likely a byproduct of the OlAm oxidation.^43,62^ It is also possible that OlAm is “activated”, *i*.*e*., the electron transfer from the amino group to Ag^+^ is enhanced when it forms a complex with the Fe ion, which enables the AgCl reduction. However, the exact appearance and reaction cycle of the catalyst require further research.

## Conclusions

The catalytic behavior of Fe ions in the formation of Ag nanocubes in nonpolar solvents was studied in detail, and an unusual dual pathway mechanism was discovered. At the beginning of the reaction, the Fe ions strongly direct the reaction of the Ag precursor toward the formation of AgCl nanoparticles rather than a thermal decomposition into icosahedral Ag (MT) nanoparticles. Subsequently, the AgCl nanoparticles are reduced by OlAm, which is also catalyzed by Fe ions. Decahedral Ag (MT) nanoparticles, which grow into nanorods, and Ag (SC) nanoparticles, which grow into nanocubes, are formed as a result of this reduction. Since the oxidation rate of the Ag (MT) nanoparticles is not affected by the Fe ions, the more the reduction rate is increased by Fe ions, the greater the proportion of Ag (MT) nanoparticles that grow into nanorods. Only an insignificant amount of iron residues was found in the resulting Ag nanocube samples. In combination with the results of experiments using different metal ions, it is hypothesized that Fe ions are specific as a catalyst for this synthesis, and Fe­(III) is likely the more effective oxidation state. The Fe ions are present in the reaction process as an organic iron complex, possibly acting as a chloride transfer agent. However, this theory could not yet be proven, and the structure of the complex could not be identified. Given the high potential for application in other chloride transfer reactions, future investigations should primarily focus on identifying the structure and reaction mechanism of the complex.

To conclude this study, a deeper understanding of the synthesis mechanisms was gained, which opens up new possibilities for optimizing the nanocube synthesis toward lower polydispersity, suppressing the formation of other nanoparticle shapes, and a wider window for particle size control. The dual function of the Fe ion catalyst is likely transferable to other metal–organic nanoparticle syntheses where chloride precursors or intermediates are used. We believe that the unusual mechanism of this catalyst qualifies it for further research into its structure and reaction cycle.

## Experimental Section

Standard glass equipment was used for all experiments performed. All equipment that came into direct contact with the reaction medium was immersed in nitric acid (65%, Fisher Scientific) prior to synthesis and subsequently rinsed with deionized (DI) water. An LTR3500 controller (Juchheim Solingen) connected to a Pt100 glass thermometer (Juchheim Solingen) was used to control the heating ramp and temperature. A Sonorex RK512H sonication bath (860 W, 35 kHz; Bandelin) was used for the redispersion of the nanoparticles. If not stated otherwise, all synthesis steps are performed under a constant argon flow (∼1 L/h).

### Materials

Silver nitrate (99.9999%), copper­(II) chloride (99.999%), iron­(III) chloride hexahydrate (≥99%), zinc chloride (99.999%), dibenzyl ether (≥98%, DBE), and oleylamine (≥98% primary amine, OlAm) were obtained from Sigma-Aldrich. Iron­(II) chloride tetrahydrate (≥99%) was purchased from Fluka, hydrochloric acid (32%) from VWR, and hexane (mixed isomers, 98+ %) from Thermo Scientific. All chemicals were used as received and without further purification.

### Synthesis Details

#### Standard Synthesis

Two precursor solutions were prepared for this hot-injection approach. The chloride precursor was prepared in a 50 mL three-neck round-bottom flask and consisted of 0.3 mL FeCl_3_·6H_2_O in DBE solution (1 g/L or 3.7 mM), 20 mL DBE, 2.07 mL OlAm, and 0.525 mL OlAmoCl in OlAm solution (1 M). The mixture was stirred vigorously (glass-covered stirrer bar 12 × 5 mm, VWR, 1300 rpm) and heated to 60 °C for the OlAmoCl to become liquid. When the temperature was stable, a vacuum was applied three times carefully. The first vacuum was held for 30 min (final value ∼0.025 mbar). The second and third vacuum was held for 15 min each (final value ∼0.02 mbar). Subsequently, the solution was heated to 260 °C at a rate of 10 K/min and maintained at this temperature until it was stable.

The silver precursor was prepared simultaneously. AgNO_3_ was dissolved in OlAm to obtain a solution with a concentration of 34.23 g/L (31.73 M). Since AgNO_3_ dissolves slowly in OlAm, the mixture was heated to 50 °C and a weak vacuum was applied (800–900 mbar). After the salt had completely dissolved, a vacuum was applied for 30 min (final value 0.025 mbar) and then repeated twice for 15 min (final value 0.025 mbar). It should be noted that the silver precursor will change color from a light yellow to a deeper yellow if it is prepared too far in advance, indicating the undesired formation of Ag seed nanoparticles.

The argon flow was stopped when both precursors reached a stable temperature. Then, 2.97 mL of the silver precursor was swiftly injected into the chloride precursor. Therefore, a 3 mL disposable syringe (Romed) connected to a stainless-steel cannula (2.0 × 200 mm, neoLab) was filled to the 2.6 mL mark (2.6 mL syringe volume +0.37 mL cannula volume). The mixture was left to react for 1 h. The heating mantle was then removed, and the mixture was cooled to 200 °C using compressed air and subsequently to 70 °C with an ice bath.

### Synthesis Variations, Particle Purification, and Characterization

Variations of the standard procedure for the studies discussed in this publication, as well as details on the particle purification and characterization methods, are given in the Supporting Information.

## Supplementary Material



## Notes

Information on synthesis variations, particle purification, and characterization; XRD data, additional photographs and SEM images, size distribution histograms, as well as a high-resolution XPS spectrum (PDF)
