# Supplementary material for: Catalysis in Silver Nanocube Formation: The Role of Iron Ions in Non-Polar Solvents
Source: ACS Nanosci Au. 2025 Oct 9;5(6):543–56. doi: 10.1021/acsnanoscienceau.5c00103 (PMC12715627; doi:10.1021/acsnanoscienceau.5c00103)
Supplement: Supplementary file 1 [file ng5c00103_si_001.pdf]

Supporting Information

Catalysis in Silver Nanocube Formation: The Role of Iron Ions in Non-Polar Solvents

Maximilian Joschko[1,2], Moritz Schattmann[1,2], Deniz Grollmusz[1,2], Tobias Reich[3], and Christina Graf*[1,2]

[1]*Hochschule Darmstadt - University of Applied Sciences, Fachbereich Chemie- und Biotechnologie, Stephanstr. 7, D-64295 Darmstadt, Germany*

[2]*EUt+ Institute of Nanomaterials & Nanotechnologies EUTINN, European University of Technology, European Union*

[3]*Johannes Gutenberg-Universität Mainz, Department of Chemistry – Nuclear Chemistry, Fritz-Strassmann-Weg 2, D-55128 Mainz, Germany*

**Corresponding author | Prof. Dr. Christina Graf | christina.graf@h-da.de*

EXPERIMENTAL SECTION

Synthesis details

Synthesis of oleylammonium chloride (OlAmoCl). OlAmoCl was synthesized using a modified procedure from Dutta *et al.*[1] A 1 M solution of OlAmoCl in oleylamine (OlAm) was obtained by adding 6 mL of hydrochloric acid to 61.08 mL OlAm, which was stirred in a 100 mL Schlenk flask under argon atmosphere. A white precipitate formed immediately. Excess water was removed from the mixture by keeping the mixture at 120 °C for 2 h under constant argon flow. The remaining water was removed by applying a vacuum (10^{-2} mbar) at 60 °C for 1 h. The mixture was cooled to ambient temperature, where it solidified, and stored as a stock solution at 8 °C in an argon atmosphere.

Samples were sent to Mikroanalytisches Labor Kolbe (Oberhausen, Germany), which verified the chloride content with ion chromatography with a Metrohm Model 930 Compact IC Flex Oven/SeS/PP/Deg after a combustion digestion in an AQF-2100H from Mitsubishi.

Nanoparticle purification. Since some organic residues in the as-received products solidify at ambient temperature and precipitate with the nanoparticles (*e.g.*, OlAmoCl), the solutions were kept at 50 °C during the purification procedure. This ensures that all excess organic matter is removed. The reaction product was filled into a 50 mL centrifugal tube and centrifuged at 800 *g* for 15 min. After removing the supernatant, 25 mL of hexane was added, and the mixture was sonicated for 5 min. The samples were centrifuged a second time at 500 *g* for 5 min, and the redispersion was carried out as in the first step. The third centrifugation step was carried out at 300 *g* for 3 min. The final redispersion was performed using 10 mL hexane, and the nanoparticles were finally stored under argon.

$FeCl_3$ variations (final product). When the influence of Fe(III) ions on the final product was studied, the synthesis was carried out with 0.625 mL OlAmoCl in OlAm solution and with a $FeCl_3 \cdot 6H_2O$ amount varying between 0.3 and 5 mg.

Since the amount of iron ions is already low in the synthesis protocol with 0.525 mmol OlAmoCl and 0.3 mg $FeCl_3 \cdot 6H_2O$, a further reduction would lead to large deviations. To extend the range of the Fe:Cl ratio to lower values, the amount of OlAmoCl was increased to 0.625 mmol. A comparison to previous studies shows that an increase in both Cl:Ag ratio and Fe ions leads to comparable results to a synthesis with a lower Cl:Ag ratio and a lower Fe content.[2]

$FeCl_3$ variations (early-stage). The studies on the influence of Fe(III) ions on the early-stage reaction were performed with 0.0, 0.3, and 0.7 mg (0.0, 1.1, and 2.6 µmol) $FeCl_3 \cdot 6H_2O$. For each concentration, a series of samples were taken after 2, 5, 15, 30, 60, and 120 s. Two samples of 1.5 mL were drawn per synthesis using a glass syringe (10 mL, Socorex Swiss) with a stainless-steel cannula (2.0x200 mm, neoLab) attached. The samples were immediately filled into ice-cooled 1.5 mL Eppendorf tubes to quench the reaction. For UV/VIS absorbance measurements, the samples were used as received. For SEM examinations, the samples were centrifuged once at 1000 *g* for 30 min, then the supernatant was discarded, and the nanoparticles were redispersed in hexane by shaking.

The analysis of these samples turned out to be rather challenging. After dilution with hexane, the unreacted precursor coagulated and precipitated along with the nanoparticles after about 2 min. The application of heat or ultrasound during the purification process likely caused the unreacted precursors to react further since the size of the AgCl particles was significantly larger (about 4 to 6 times) in the SEM compared to AgCl particles from the same sample when it was not exposed to heat or ultrasound. Therefore, the samples were centrifuged once at low acceleration and redispersed with hexane by shaking to remove at least a large part of the long-chain organic compounds. Immediately after redispersion, the samples were applied to a copper grid. However, examination in the TEM was not possible due to the presence of organic residues and the too rapid decomposition of the AgCl particles under the electron beam. Examination in the SEM was possible, but the resolution suffered from the aforementioned problems. Ag and AgCl particles could be distinguished because AgCl began to decompose in the electron beam, but Ag did not.

Metal ion variations. Various metal ions were introduced into the standard synthesis instead of Fe(III). 1.1 µmol of $FeCl_2 \cdot 4H_2O$, $CuCl_2 \cdot 2H_2O$, $ZnCl_2$ (0.22 mg, 0.19 mg, or 0.15 mg, respectively), or no additional metal ions were added to the reaction mixture. The $FeCl_2 \cdot 4H_2O$ was also dissolved in DBE, while $CuCl_2 \cdot 2H_2O$ and $ZnCl_2$ were weighed in directly due to their poor solubility in DBE.

Characterization

Thermogravimetric analysis (TGA). The purified nanoparticles were analyzed for organic residues using a Netzsch TG209 F1 libra with the external cooling system Julabo F32-MA. 2-6 mg dried sample was used for each analysis and measured in an alumina crucible (85 µL, Netzsch). After a 10 min equilibration period, the system was heated to 600 °C at a rate of 10 K/min. A nitrogen gas flow of 20 mL/min was used during the measurement.

Scanning electron microscopy (SEM). A Hitachi SU 5000 was used to examine the nanoparticles. The microscope was operated in the secondary electron (SE) mode with an electron acceleration voltage of 15 kV and a spot intensity of 30. The working distance was set to 3 mm. The undiluted nanoparticle dispersion (as received from the purification process) was dropped onto an uncoated copper grid (Cu 400 mesh, Plano GmbH) and dried before the measurement. The images were analyzed using the FIJI software. To estimate the particle size, at least 300 particles per synthesis on three or more different images were evaluated.

To determine the number percentage of multiply twinned Ag nanoparticles, 5000-10000 particles were analyzed on 5-8 different images. The uncertainties were estimated by calculating standard deviations from these images. The procedure was described in a previous publication.[2]

Transmission electron microscopy (TEM). TEM investigations were conducted on a Zeiss EM 109 with 80 kV operation voltage. The undiluted nanoparticle dispersion (as received from the purification process) was dropped onto a carbon-coated copper grid (carbon-coating type A, 6-10 nm thickness, Cu 400 mesh, Plano GmbH) and dried before the measurement. The evaluation was carried out as described above.

Energy-dispersive X-ray analysis (EDX). The samples were examined for their elemental composition using an EDAX X-ray detector (Octane Elect Plus), with which the SEM was equipped. The SEM was operated at an acceleration voltage of 15 kV and a spot intensity of 50 with the working distance set to 10 mm. The resolution of the detector was 126.2 eV. A thick layer (several micrometers) of the sample was deposited on an uncoated copper grid and measured on a STEM holder. Three different areas of 2x2 mm^2 were examined with 200 s accumulation time. The results for chlorine and silver were corrected by measurements with standards as suggested by EDAX.[3]

UV/VIS absorbance spectroscopy. The UV/VIS absorbance measurements were carried out in a Cary 5000 UV-VIS-NIR spectrometer from Agilent Technologies. The unpurified samples (s. $FeCl_3$variations (early-stage)) were diluted 30-fold with hexane and immediately measured to ensure comparability and that the samples remained unchanged. The measurements were carried out in special quartz cuvettes (type 26.715/Q/10/Z20; Starna) and directly measured in the range of 350 to 800 nm.

X-ray diffraction spectrometry (XRD). A Bruker D8 Eco diffractometer was used, equipped with a LYNXEYE XE-T detector and a Cu $K_{\alpha 1}$ radiation source (25 kV, 40 mA). The wavelength of Cu $K_{\alpha 1}$ radiation is 0.15405 nm. The angular range of the measurements was 25-80° 2θ with a step size of 0.025°. 2-3 mg of silver nanoparticles were dried onto a glass slide (76x26 mm, Elka) and then measured in Bragg-Brentano geometry with a fixed sample stage and movable X-ray source and detector arm. The samples were measured right after sample purification.

Atomic absorption spectroscopy (AAS). The silver contents in the final nanoparticle samples were determined by flame AAS. The model used was an iCE 3000 from Thermo Scientific.

Silver samples were prepared by drying ~5 mg of the nanoparticles in an inert atmosphere, dissolving the dried nanoparticles in nitric acid, and diluting the resulting digest to estimate Ag concentrations of 2-10 mg/L with 1.4 % nitric acid content. The samples were each measured three times at an emission wavelength of 328.1 nm with a D2 correction in an air-acetylene flame.

Inductively coupled plasma optical emission spectroscopy (ICP-OES). The iron contents in the final nanoparticle samples were determined by Mikroanalytisches Labor Kolbe (Oberhausen, Germany). After a microwave digestion in a MARS 6 from CEM, the samples were analyzed in a Spectro Arcos ICP-OES from Spectro.

X-ray photoelectron spectroscopy (XPS). The XPS measurements were performed using a spectrometer from SPECS Surface Nano Analysis GmbH, which was equipped with a non-monochromatized Al/Mg K_α X-ray source and a PHOIBOS 100 MCD energy analyzer. The powdered sample was pressed into indium foil under Ar atmosphere in a glove box ($\leq$ 1 ppm O_2). Then, the sample was transferred into the XPS spectrometer under N_2 atmosphere to avoid direct contact with air. The survey spectrum and multiple scans of the Ag 3d and C 1s lines were recorded with analyzer pass energies of 50 eV and 13 eV, respectively. The pressure in the vacuum chamber was $\leq 5 \cdot 10^{-9}$ mbar. Binding energies were determined using the C 1s binding energy of 285.0 eV as reference. The data analysis was done with the CasaXPS software. The molar concentration of Cl was estimated based on the Cl 2p and Ag 3d lines. The atomic ratios were calculated using the theoretical photoionization cross sections.

ADDITIONAL DATA

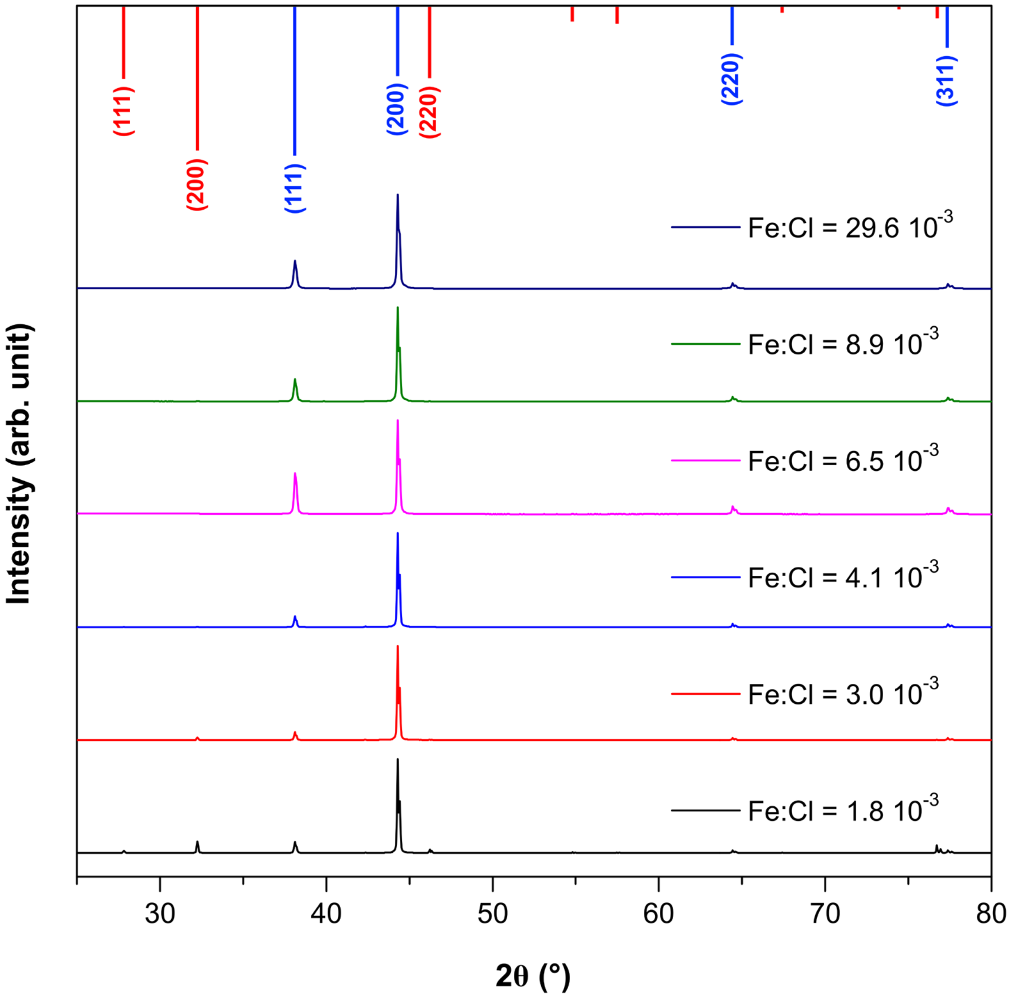

Figure S1: X-ray diffractograms of Ag nanocubes, which were prepared with an increasing amount of Fe(III) ions (increasing Fe:Cl ratio) added. The diffraction patterns of AgCl (COD: 9011666) and of Ag (COD: 9008459) are displayed on the top of the image in red and blue, respectively.

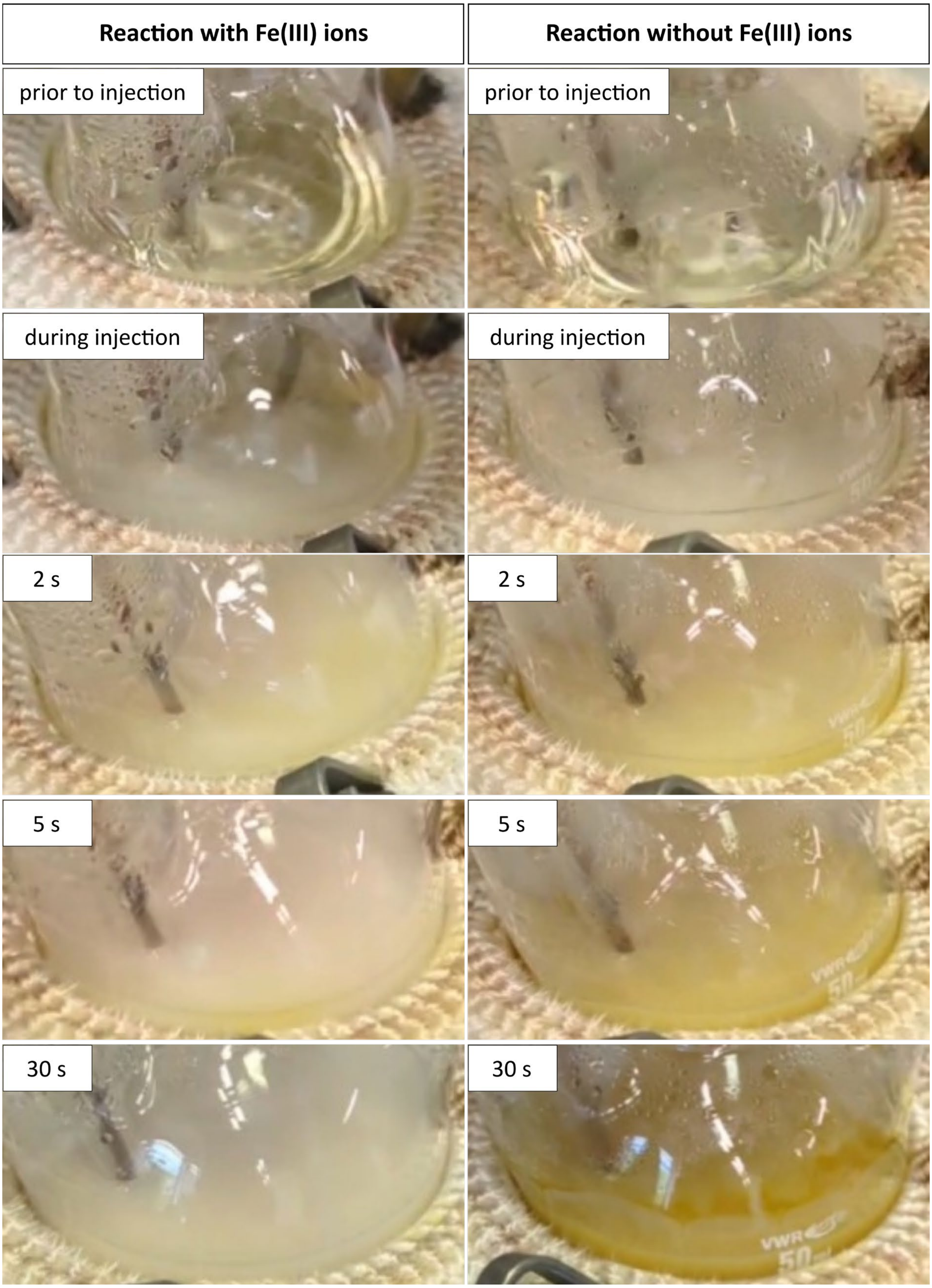

Figure S2: Color changes of the reaction mixture without the addition (left) and with the addition of 1.1 µmol Fe(III) ions (right) over the first 30 s of the reaction.

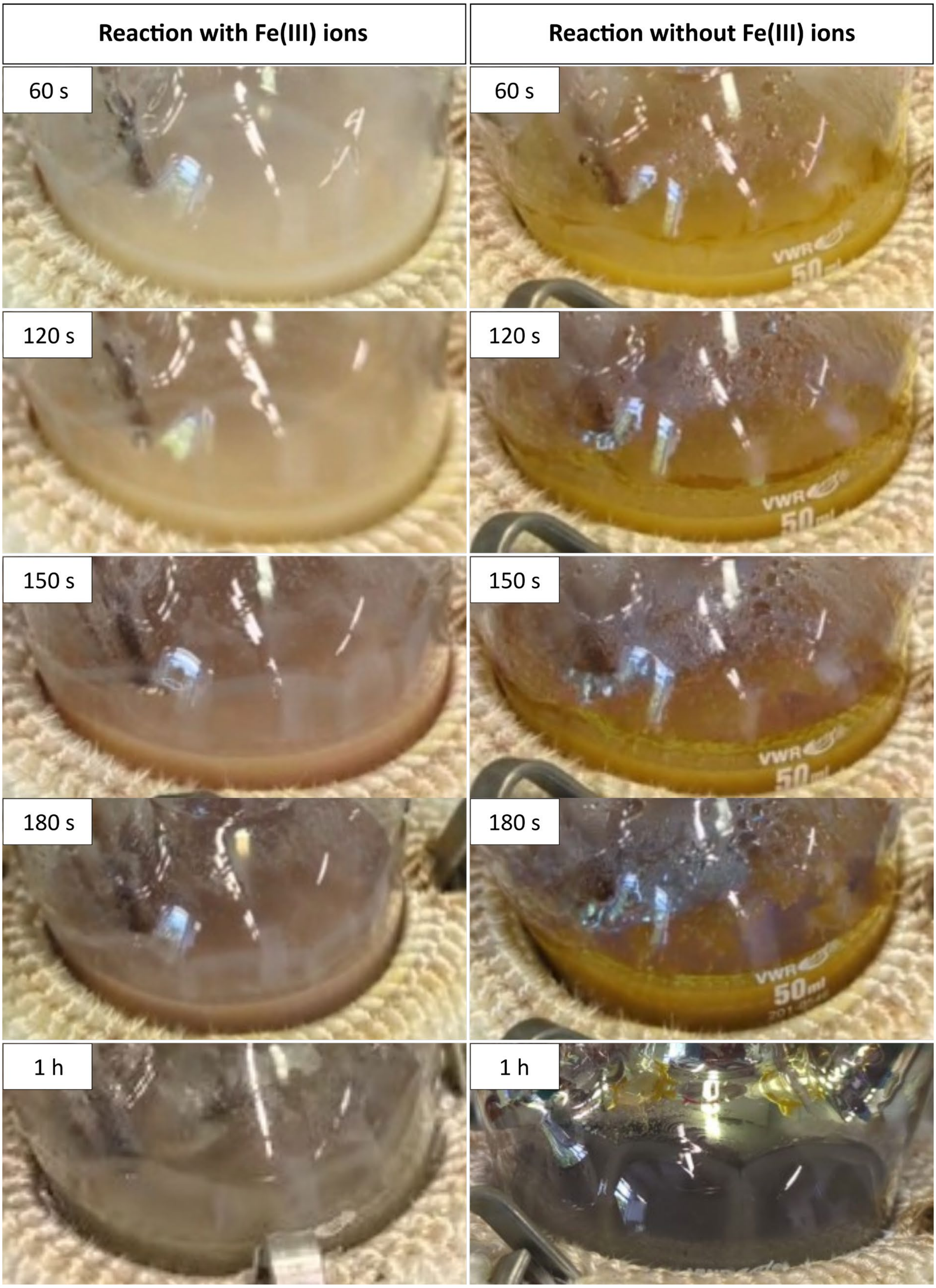

Figure S3: Color changes of the reaction mixture without the addition (left) and with the addition of 1.1 µmol Fe(III) ions (right) from 60 s to the end of the reaction.

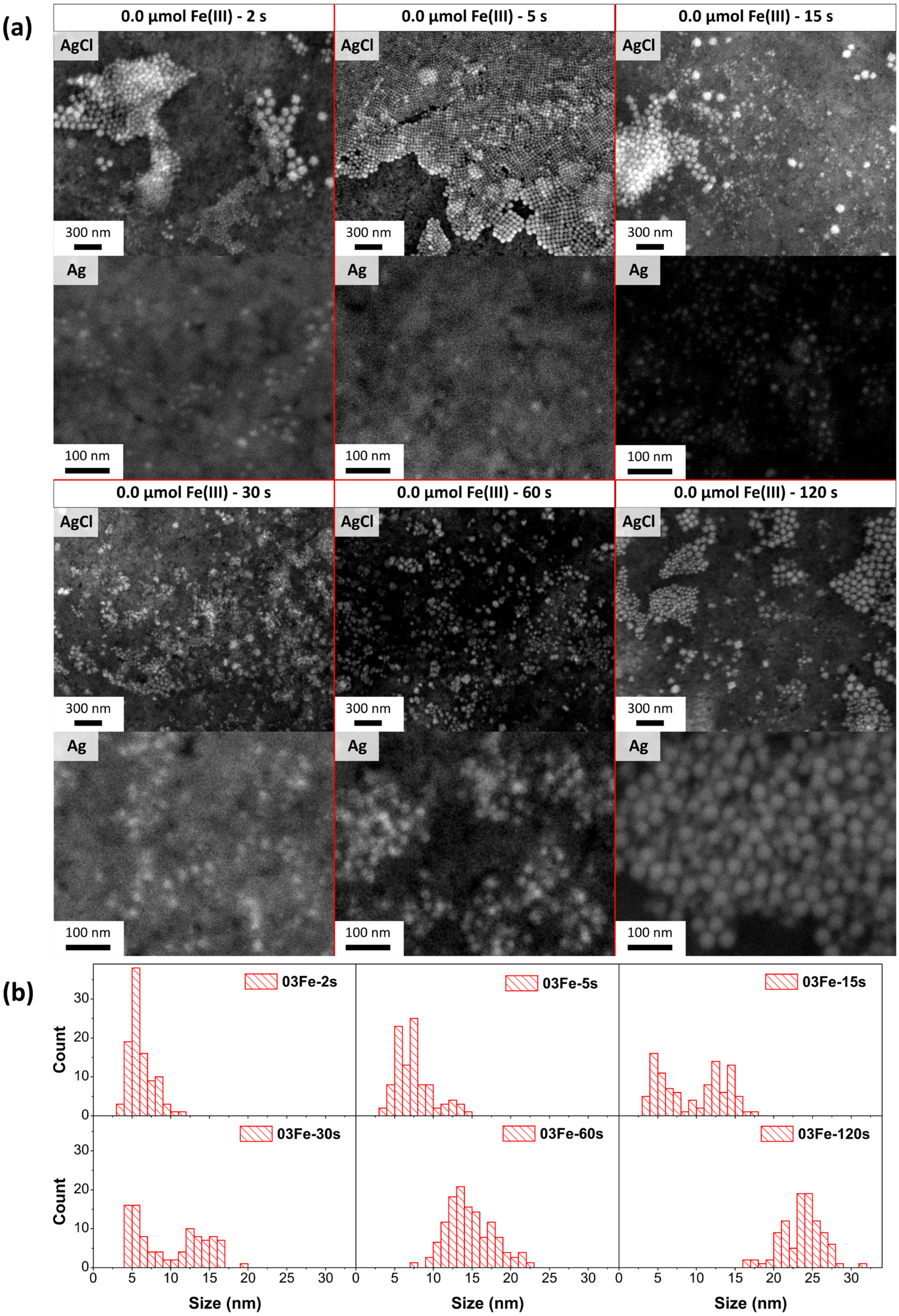

Figure S4: (a) SEM images of the AgCl and Ag nanoparticles from the experiment with 1.1 µmol Fe(III) from 2 to 120 s, as well as (b) the histograms of the Ag nanoparticles.

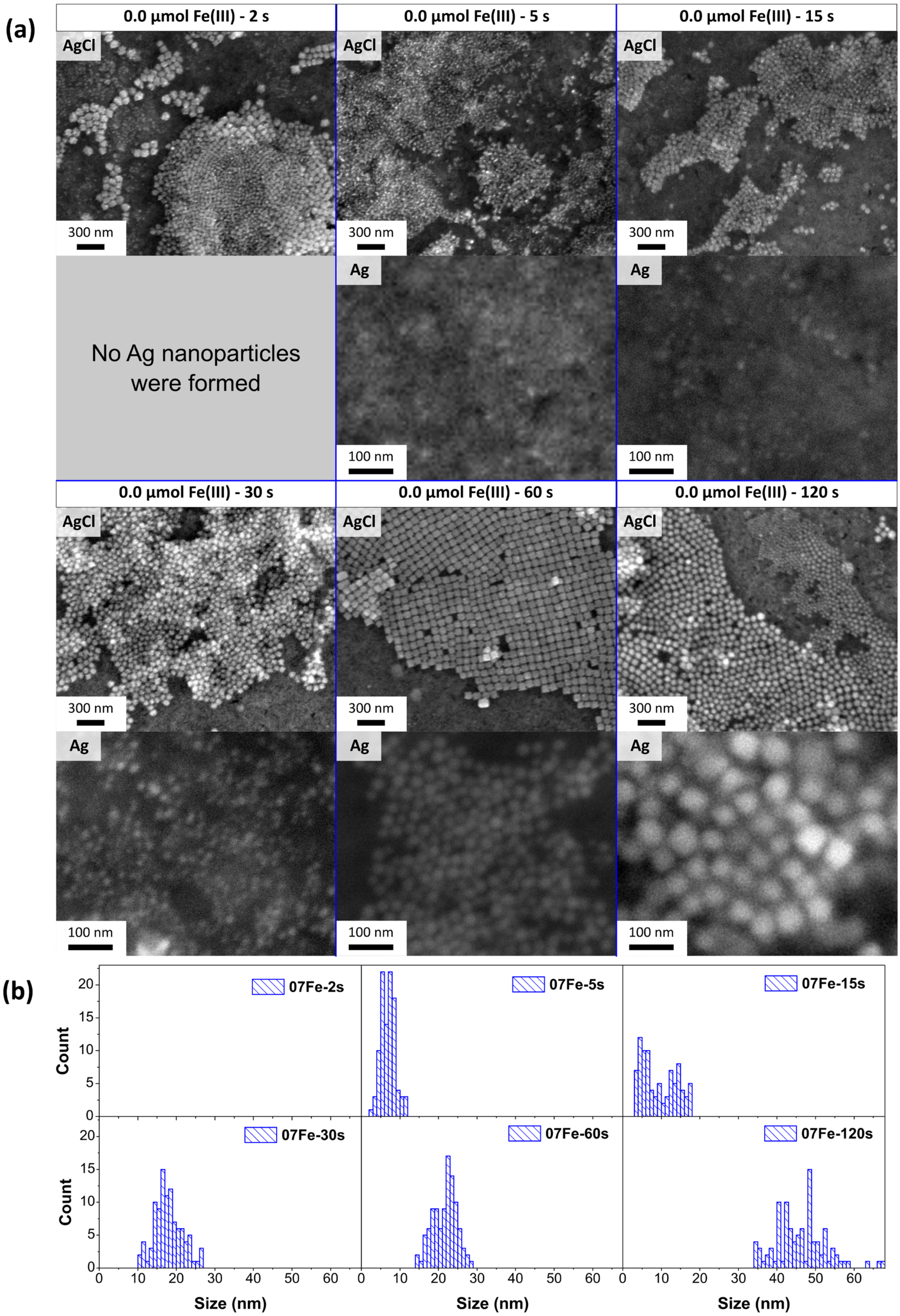

Figure S5: (a) SEM images of the AgCl and Ag nanoparticles from the experiment with 2.6 µmol Fe(III) from 2 to 120 s, as well as (b) the histograms of the Ag nanoparticles.

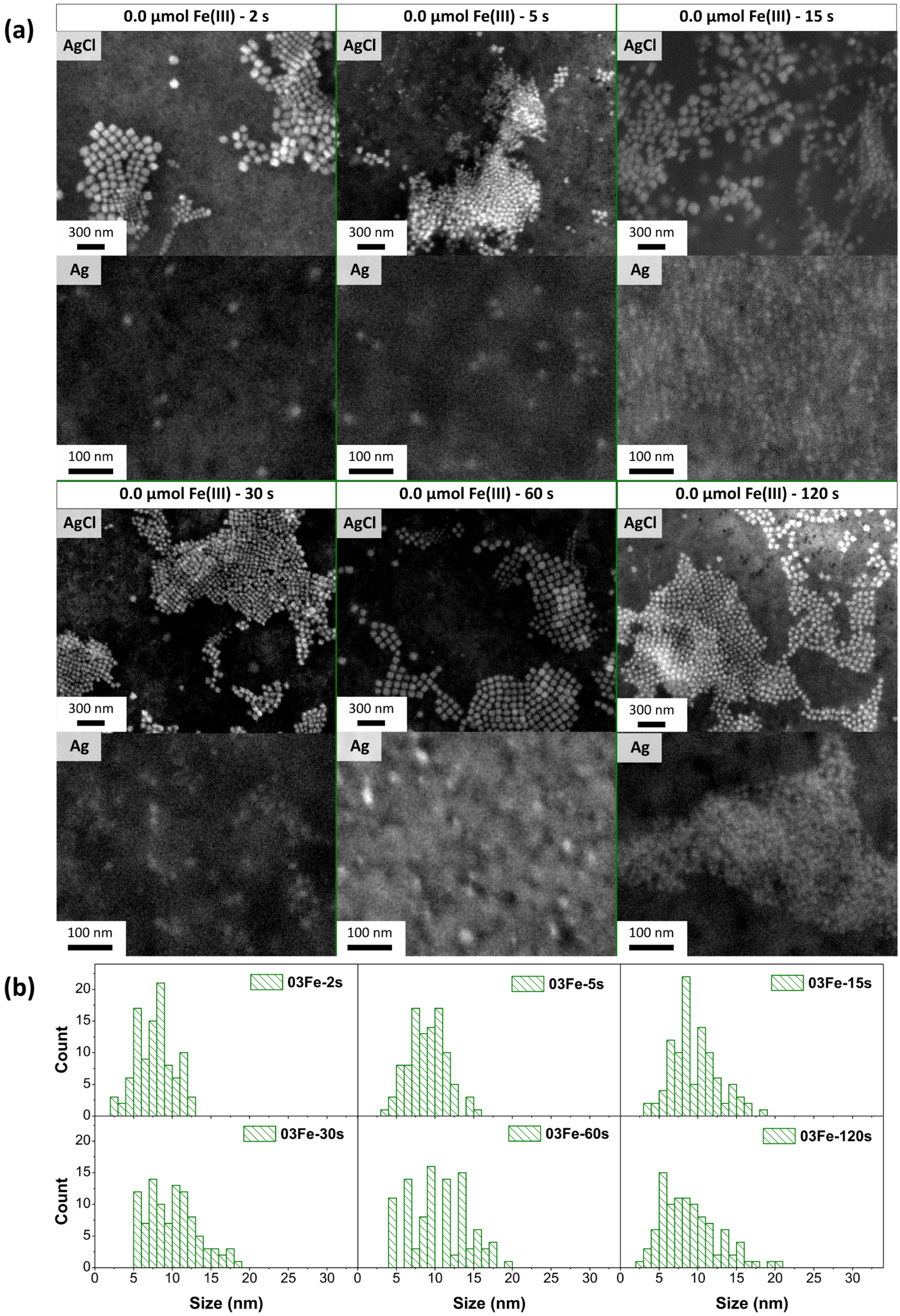

Figure S6: (a) SEM images of the AgCl and Ag nanoparticles from the experiment without Fe(III) from 2 to 120 s, as well as (b) the histograms of the Ag nanoparticles.

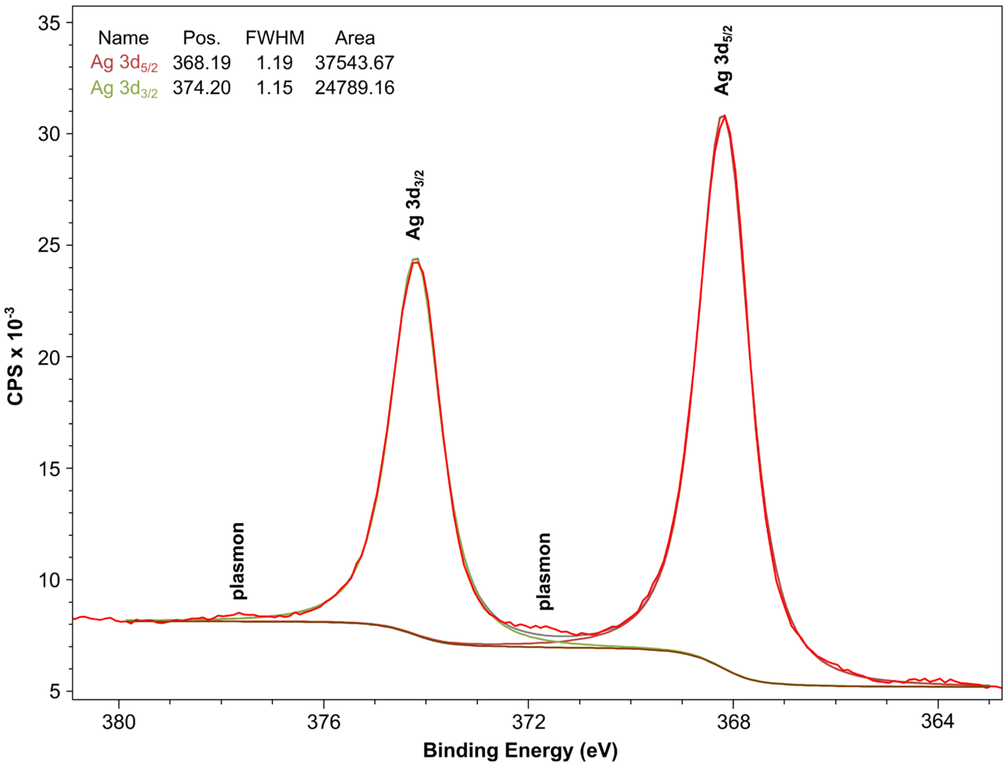

Figure S7: Ag 3d XPS spectrum of purified Ag nanocubes recorded with non-monochromatized Al K_α radiation (1486.6 eV) and 13 eV analyzer pass energy. For peak fitting, the Shirley background and symmetric peak shapes were used. The electron binding energies of the Ag $3d_{5/2}$ and Ag $3d_{3/2}$ peaks are 368.2 and 374.2 eV, respectively. The small satellite peaks at ~371.8 and ~377.8 eV, respectively, are assigned to plasmon losses. Both, the positions of the Ag 3d lines as well as the two satellite peaks, are indicative for metallic Ag.[4,5]

REFERENCES

(1) Dutta, A.; Behera, R. K.; Dutta, S. K.; Adhikari, S. D.; Pradhan, N. Annealing $CsPbX_3$ (X = Cl and Br) Perovskite Nanocrystals at High Reaction Temperatures: Phase Change and Its Prevention. *J. Phys. Chem. Lett.* **2018**, *9* (22), 6599–6604. DOI: 10.1021/acs.jpclett.8b02825. Published Online: Nov. 6, 2018.

(2) Joschko, M.; Schattmann, M.; Grollmusz, D.; Graf, C. Controlling the formation of fast-growing silver nanocubes in non-polar solvents. *Nanoscale* **2025**, *17* (24), 14727–14740. DOI: 10.1039/D5NR01350J. Published Online: Jun. 19, 2025.

(3) Mausz, J., EDAX AMTEK, personal communication, **2024**.

(4) Johansson, G.; Hedman, J.; Berndtsson, A.; Klasson, M.; Nilsson, R. Calibration of electron spectra. *J. Electron Spectrosc. Relat. Phenom.* **1973**, *2* (3), 295–317. DOI: 10.1016/0368-2048(73)80022-2.

(5) Leiro, J.; Minni, E.; Suoninen, E. Study of plasmon structure in XPS spectra of silver and gold. *J. Phys. F: Met. Phys.* **1983**, *13* (1), 215–221. DOI: 10.1088/0305-4608/13/1/024.
